# The cGAS-STING pathway in ovarian cancer: from genomic instability to immunotherapeutic vulnerability

**DOI:** 10.3389/fimmu.2026.1977503

**Published:** 2026-10-05

**Authors:** Hui-Hui Chen, Feng-Xian Ni, Dong-Hui Huang, Ze-Bo Jiang

**Affiliations:** 1 Zhuhai Hospital of Integrated Traditional Chinese and Western Medicine, Zhuhai, Guangdong, China; 2 Zhuhai Hospital Affiliated to Faculty of Chinese Medicine, Macau University of Science and Technology, Zhuhai, Guangdong, China

**Keywords:** cGAS-STING, DNA damage response, genomic instability, immune checkpoint blockade, innate immunity, ovarian cancer, PARP inhibitors, tumor microenvironment

## Abstract

Ovarian cancer remains the most lethal gynecologic malignancy, a status largely attributable to late-stage diagnosis and inevitable chemoresistance. The cGAS-STING pathway, a cornerstone of innate immune surveillance, has emerged as a critical nexus linking genomic instability to antitumor immunity—a connection of particular relevance in ovarian cancer, where chromosomal instability is a defining hallmark. This review synthesizes current understanding of the cGAS-STING pathway in ovarian cancer, spanning molecular mechanisms to translational opportunities. We delineate the dualistic nature of STING signaling, which orchestrate robust T cell-mediated antitumor immunity under certain conditions yet paradoxically foster immunosuppression and tumor progression in others. Particular emphasis is placed on the intricate interplay between DNA damage repair deficiencies—especially in *BRCA1/2* and homologous recombination—and STING-dependent immune activation, which forms the mechanistic basis for the immunomodulatory effects of PARP inhibitors. We critically examine the TME, where CAFs and myeloid cells integrate STING signals to either potentiate or constrain antitumor immunity. The landscape of STING-modulating therapeutic strategies is surveyed, encompassing direct agonists, nanoparticle-based delivery platforms, and rational combinations with immune checkpoint blockade and DNA-damaging agents. Finally, we identify key unresolved questions, including the need for predictive biomarkers, strategies to overcome resistance, and the challenge of balancing therapeutic efficacy against potential toxicities. By integrating recent discoveries with established paradigms and explicitly addressing the “double-edged sword” duality of STING signaling, the stromal response, and a proposed roadmap for biomarker-guided therapeutic development, this review aims to provide a framework that distinguishes itself from prior comprehensive reviews by integrating recent discoveries with established paradigms and explicitly addressing the ‘double-edged sword’ duality of STING signaling, the stromal response, and a proposed roadmap for biomarker-guided therapeutic development.

## 1 Introduction

Ovarian cancer, particularly high-grade serous ovarian cancer (HGSOC), remains the leading cause of gynecologic cancer mortality (1). Despite initial responses to debulking surgery and platinum-based chemotherapy, recurrence is near-inevitable, and each successive line of therapy yields diminishing returns (2–4). The five-year survival rate for advanced-stage disease has seen only marginal improvement over decades, underscoring an urgent need for novel therapeutic paradigms (5). While PARP inhibitors (PARPi) have exploited synthetic lethality in BRCA1/2-mutant tumors (6), resistance inevitably emerges. Similarly, immune checkpoint blockade (ICB), which has revolutionized other malignancies, show disappointingly modest efficacy in unselected ovarian cancer populations (7, 8).

Over the past five years, several comprehensive reviews have addressed the cGAS-STING pathway in ovarian and gynecologic cancers, providing foundational summaries of STING biology, DNA damage signaling, and therapeutic opportunities (6–9). However, the field has evolved rapidly, with three paradigm-shifting developments that warrant a new synthesis. First, the discovery of the STING-WNT-POSTN axis has fundamentally revised our understanding of how cancer cell genomic instability communicates with the tumor stroma, establishing CAFsas central mediators of STING-dependent immunosuppression and PARP inhibitor resistance (10). Second, recent single-cell and spatial transcriptomic studies have revealed striking spatial and temporal heterogeneity in STING activation across ovarian cancer metastatic sites, with important implications for biomarker assessment and therapeutic targeting (11). Third, the mixed results of recent clinical trials combining PARP inhibitors with immune checkpoint blockade have prompted a critical re-evaluation of the assumptions underlying these combinations, highlighting the need for biomarker-driven patient selection and rational sequencing (12, 13). Our review distinguishes itself from prior work by integrating these recent advances into a unified conceptual framework that explicitly addresses the “double-edged sword” duality of STING signaling, the critical role of the stromal response, and a proposed roadmap for biomarker-guided therapeutic development.

The advent of poly(ADP-ribose) polymerase inhibitors (PARPi) represented a genuine breakthrough, exploiting the synthetic lethality between PARP inhibition and homologous recombination repair (HRR) deficiency—a defect present in approximately half of HGSOC cases due to germline or somatic mutations in BRCA1/2 or other HRR pathway components (14–16). Yet even here, resistance inevitably develops, and the search for strategies to extend and enhance PARPi efficacy continues unabated. More recently, ICB has revolutionized treatment paradigms across multiple tumor types, but its impact in ovarian cancer has been disappointingly modest, with response rates in unselected populations hovering below 15% (17, 18). This relative immunotherapy resistance has prompted intense investigation into the barriers to effective antitumor immunity in ovarian cancer and the strategies that might overcome them.

It is at this intersection—between DNA damage, innate immune sensing, and adaptive antitumor immunity—that the cGAS-STING pathway has emerged as a subject of extraordinary interest (19). The cyclic GMP-AMP synthase (cGAS)-stimulator of interferon genes (STING) pathway constitutes a primary surveillance mechanism for aberrant cytosolic DNA, orchestrating a type I interferon (IFN-1) response that bridges innate and adaptive immunity (20). Mechanistically, cGAS binds cytosolic double-stranded DNA (dsDNA) and produces 2’3’-cyclic GMP-AMP (cGAMP), which activates STING to recruit TANK-binding kinase 1 (TBK1) and phosphorylate interferon regulatory factor 3 (IRF3) (21, 22). This cascade promotes dendritic cell (DC) maturation and CD8^+^ T cell cross-priming through type I IFN and chemokine secretion (23, 24). In ovarian cancer, the convergence of frequent genomic instability, inherent or therapy-induced DNA damage, and an immunologically complex TME creates a unique context in which STING signaling assumes particular importance (25).

The therapeutic implications are profound. STING activation can potentiate antitumor immunity through multiple mechanisms: direct effects on tumor cells that enhance their immunogenicity, activation of DCs and cross-presentation of tumor antigens, reprogramming of tumor-associated macrophages toward pro-inflammatory phenotypes, and sensitization of tumors to ICB (26, 27). Yet the pathway also harbors darker dimensions—chronic STING activation can drive immunosuppressive programs, promote metastasis through non-canonical NF-κB signaling, and contribute to PARPi resistance through complex stromal interactions (28, 29). Understanding when and how STING signaling tips the balance between immunity and tolerance is therefore essential for therapeutic success.

This review aims to provide a comprehensive synthesis of current knowledge regarding the cGAS-STING pathway in ovarian cancer, spanning molecular mechanisms, TME interactions, and therapeutic opportunities. We begin by establishing the fundamental biology of the pathway, emphasizing its unique features in the context of ovarian cancer. We then explore the intricate relationships between DNA damage repair deficiencies and STING activation, with particular attention to the immunomodulatory effects of PARPi. The TME section examines how STING signals emanating from cancer cells influence stromal and immune populations, creating complex feedback loops that may either promote or suppress antitumor immunity. Therapeutic strategies are critically evaluated, including direct STING agonists, nanoparticle delivery platforms, combination regimens, and the emerging concept of STING inhibition. We conclude by identifying key challenges and proposing a roadmap for future investigation. Throughout, we emphasize the dualistic nature of STING signaling and the importance of context in determining therapeutic outcomes.

## 2 The cGAS-STING pathway: molecular architecture and functional logic

### 2.1 cGAS: The cytosolic DNA sensor

cGAS is a nucleotidyltransferase that binds double-stranded DNA in a sequence-independent manner, with recognition governed instead by DNA length and structure (14). Upon DNA binding, cGAS undergoes a conformational rearrangement that activates its catalytic domain, enabling the synthesis of the second messenger 2’3’-cGAMP from ATP and GTP (30). This enzymatic activity is exquisitely regulated: DNA binding induces dimerization and oligomerization, and the catalytic efficiency is strongly influenced by DNA length, with longer DNA fragments promoting more robust cGAMP production.

The subcellular localization of cGAS bears directly on ovarian cancer biology. Although canonically cytoplasmic, cGAS can localize to the nucleus, where it is tethered to chromatin and its activity is suppressed to prevent inappropriate activation by genomic DNA (31–33). Nuclear cGAS is maintained in an inactive state through interactions with histones, particularly the H2A-H2B dimer, and this tethering can be disrupted by chromatin remodeling or DNA damage—events that occur frequently in the genomically unstable milieu of ovarian cancer (33). The dynamic equilibrium between nuclear and cytoplasmic cGAS thus represents a critical checkpoint governing the threshold for innate immune activation.

Sources of cytosolic DNA that trigger cGAS in ovarian cancer cells are diverse, including micronuclei generated by mitotic errors, cytoplasmic chromatin fragments from senescent cells, mitochondrial DNA released during stress, and exogenous DNA from viral infection or therapeutic nanoparticles (34–37). The genomic instability characteristic of ovarian cancer ensures a steady supply of these DNA species, providing a constant low-level stimulus that may, depending on its magnitude and context, contribute to either immune surveillance or immune exhaustion. Recent single-cell studies of HGSOC have elegantly resolved this paradox: while chromosomal instability does trigger cGAS-STING pathway activation and inflammatory signaling, this response is strictly confined to tumors that remain predominantly diploid. By contrast, tumors that have undergone whole-genome doubling (WGD), despite exhibiting higher rates of chromosomal missegregation and micronucleation, show *STING1* repression and immunosuppressive phenotypes (11). Beyond this WGD-mediated switch, specific genetic drivers of genomic instability also shape the outcome—*CDK1*2 loss induces DNA2 accumulation, replication stress, cytosolic dsDNA accumulation, and cGAS-STING activation (38). However, when STING signaling becomes chronic, it drives immune exhaustion through PD-L1 upregulation and accumulation of regulatory T cells (Tregs) and myeloid-derived suppressor cells (MDSCs) (11, 21, 38). This duality recurs throughout the biology of the pathway and represents a central challenge for therapeutic manipulation.

### 2.2 STING: signal integration and downstream effectors

STING, encoded by *TMEM173*, functions as the central signal integrator. The protein resides predominantly in the endoplasmic reticulum (ER) as a dimer in an autoinhibited conformation. cGAMP binding to STING induces a dramatic conformational change, triggering its translocation from the ER via the ER-Golgi intermediate compartment (ERGIC) to perinuclear puncta where downstream signaling ensues (39). This trafficking is essential for signal propagation and is regulated by a complex interplay of palmitoylation, ubiquitination, and interaction with trafficking factors.

The canonical downstream cascade involves recruitment of TBK1, which phosphorylates STING at its C-terminal tail, creating a docking site for IRF3. TBK1 subsequently phosphorylates IRF3, promoting its dimerization, nuclear translocation, and transcriptional activation of type I interferon genes (IFNβ and IFNα subtypes) and a broad array of interferon-stimulated genes (ISGs) (40–42). This transcriptional program establishes an antiviral and antitumor state that includes upregulation of major histocompatibility complex (MHC) class I molecules, chemokines such as CXCL9 and CXCL10 that recruit T cells, and various effector molecules that enhance immune recognition (43, 44).

Yet STING signaling is not monolithic. Beyond the canonical TBK1-IRF3 axis, STING activates NF-κB through both canonical and non-canonical pathways, inducing a distinct transcriptional program rich in inflammatory cytokines such as TNF, IL-1β, and IL-6 (45, 46). The balance between IRF3 and NF-κB signaling is modulated by the duration and intensity of STING activation, the specific cellular context, and engagement of feedback regulatory loops (46). In ovarian cancer, where chronic low-grade inflammation often characterizes the TME, the NF-κB arm of STING signaling may predominate, promoting an immunosuppressive milieu that subverts antitumor immunity (47, 48). This contextual variability underscores the importance of understanding STING signaling in the specific cellular and microenvironmental setting of ovarian cancer.

A critical unresolved question concerns the biological determinants that dictate whether STING activation promotes immunity or tolerance. Several factors appear to be involved: the duration of STING activation, with acute signaling promoting type I IFN production and chronic signaling driving NF-κB-dependent inflammatory programs; the specific cellular context, with STING activation in cancer cells, DCs, CAFs, and macrophages producing divergent outcomes; and the balance between IRF3 and NF-κB signaling, which determines the transcriptional program induced. This heterogeneity has important implications for therapeutic development, as it suggests that a “one-size-fits-all” approach to STING modulation is unlikely to be successful. This concept will be referenced throughout subsequent sections, and readers are directed to this section for the foundational mechanistic explanation. The molecular architecture and regulatory networks of the cGAS-STING pathway are summarized in **Figure 1**.

**Figure 1:**
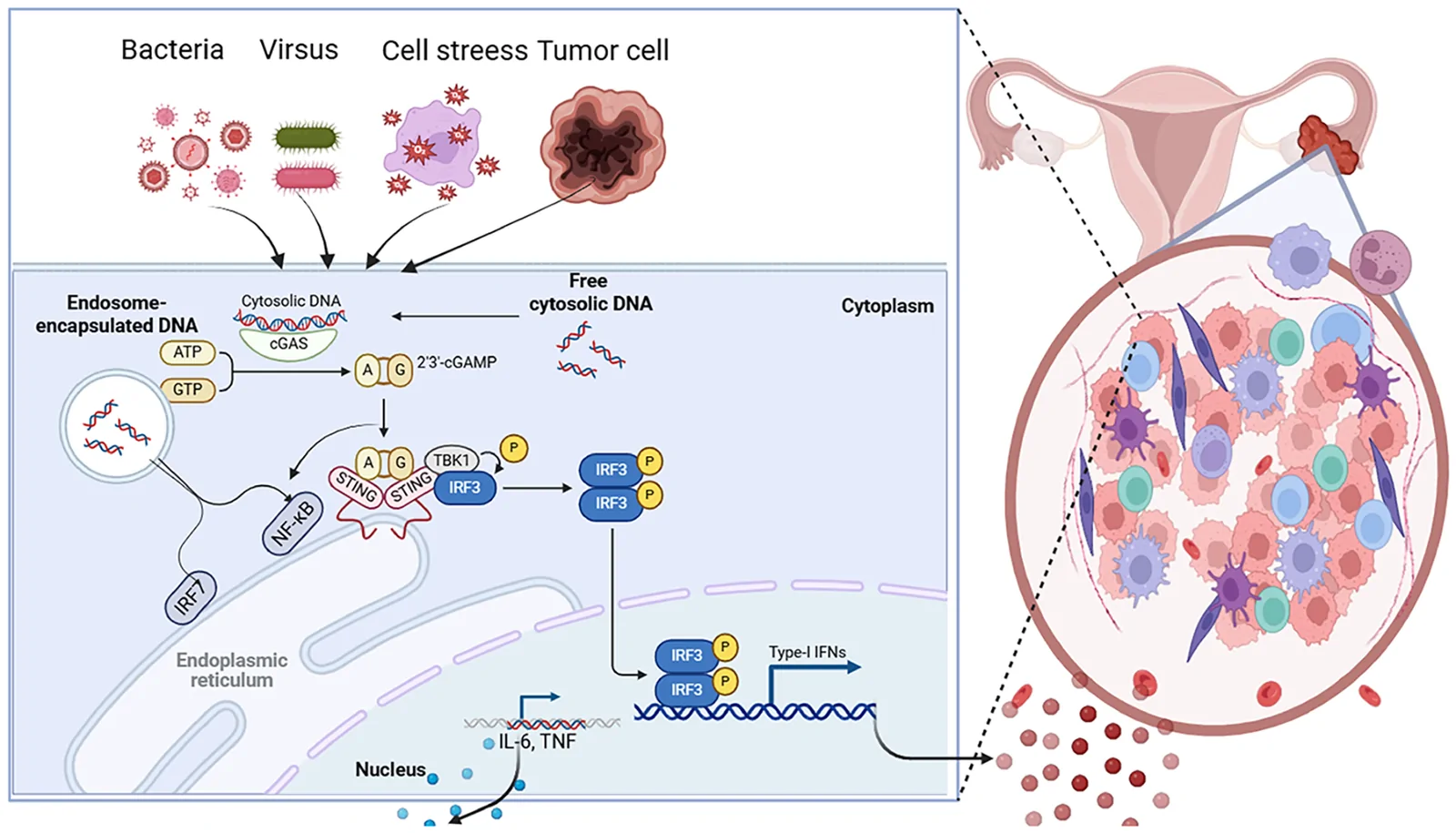
Molecular architecture and regulatory networks of the cGAS-STING pathway. Cytosolic dsDNA is bound by cGAS, producing 2’3’-cGAMP, which activates STING at the ER. STING translocates via ERGIC to the Golgi, recruits TBK1, and phosphorylates IRF3 and NF-kB. These transcription factors drive type I interferon, ISG, and pro-inflammatory cytokine expression, bridging innate and adaptive immunity. STING signaling is tightly controlled by positive and negative regulators. Negative regulators include TRIM27, USP35, and PPT1, which promote STING degradation or suppress activity, and epigenetic repressors such as SETDB1-TRIM28 that dampen pathway components. Positive regulators including ZNFX1 and CENPM amplify signaling. Disruption of this balance in ovarian cancer contributes to either immune evasion or chronic inflammation-driven tumor progression.

### 2.3 Pathway termination and homeostasis

Equally critical are the mechanisms that restrain STING activity, preventing excessive inflammation that could prove detrimental to the host. Multiple layers of negative regulation operate at distinct levels. At the level of cGAS, post-translational modifications including ubiquitination, sumoylation, and acetylation modulate its DNA binding and enzymatic activity. The deubiquitinating enzyme USP14 has been shown to remove K48-linked ubiquitin chains from cGAS, preventing its degradation, while other deubiquitinases such as USP27X can similarly deubiquitinate cGAS to enhance its activity (48, 49). The net effect of these opposing modifications determines the amplitude of cGAS activation.

STING itself is subject to stringent negative regulation, primarily through degradation pathways. Following activation, STING undergoes K48-linked polyubiquitination, targeting it for proteasomal degradation—a process that limits the duration of signaling and prevents persistent inflammation. The E3 ubiquitin ligases mediating this degradation include RNF5 (targeting K150), TRIM30α (targeting K275), and TRIM29 (targeting K370), among others. The physiological importance of this regulatory axis is underscored by the finding that TRIM21 deficiency leads to STING accumulation and heightened type I IFN responses in autoimmune models (50–52), while the deubiquitinase CYLD antagonizes this process by removing K48-linked chains from STING, thereby sustaining signaling (53). Recent work has further identified the BAG2-STUB1 complex as a regulator of STING stability in cancer models, with STUB1 catalyzing K48-linked ubiquitination at K338 and K370 (52). Additionally, STING degradation via the autophagy-lysosomal pathway has been described, providing a parallel mechanism for signal termination (54–56). The balance between activating and inhibitory ubiquitination events, mediated by distinct E3 ubiquitin ligases and deubiquitinases, fine-tunes the cellular response to STING activation (51, 52). In ovarian cancer, disruption of these regulatory mechanisms may contribute to sustained STING signaling and its associated pathological consequences.

Two aspects of pathway regulation deserve particular emphasis in the context of ovarian cancer. STING trafficking from ER to Golgi is required for signaling and represents a therapeutic vulnerability. In HGSOC, N-MYC overexpression suppresses STING oligomerization, impairing signaling and leading to an immunologically ‘cold’ TME (42). In low-grade serous ovarian cancer, STIM1 overexpression sequesters STING in the ER, attenuating IFN production (43). Importantly, these defects create a therapeutic vulnerability—ovarian cancer cells with impaired STING signaling show heightened sensitivity to oncolytic viruses (57), and pharmacologic inhibition of STING in CAFs sensitizes ovarian tumors to platinum chemotherapy (58). These observations collectively underscore the therapeutic potential of modulating STING trafficking and activity in ovarian cancer (59). Conversely, facilitating STING trafficking represents a potential strategy for enhancing innate immune activation. Second, downstream effector pathways exhibit cell-type specificity. In cancer cells, STING activation may promote apoptosis or senescence (60–62); in CAFs, it may drive immunosuppressive programs; in myeloid cells (44), it may promote pro-inflammatory or anti-inflammatory phenotypes depending on the activation context. Understanding this cell-type specificity is essential for rational therapeutic design.

## 3 Genomic instability and DNA damage: the substrate for STING activation

### 3.1 The genomically unstable landscape of ovarian cancer

HGSOC stands among the most genomically unstable of all human cancers. This instability manifests at multiple levels—chromosomal, structural, and mutational—and provides a constant source of the aberrant DNA species that activate the cGAS-STING pathway (5). Pervasive chromosomal instability (CIN) characterizes the majority of HGSOC tumors (63), with TP53 mutations present in virtually all cases and extensive copy number alterations affecting numerous genomic regions (64, 65). This CIN generates micronuclei and chromatin fragments in the cytoplasm, providing a constant low-level stimulus for cGAS (14, 66, 67).

The origins of this instability are multifactorial. Defects in homologous recombination repair (HRR), present in approximately 50% of HGSOC due to mutations in *BRCA1*, *BRCA2*, or other HRR genes, represent a primary driver of genomic instability (68–70). Loss of HRR leads to reliance on error-prone repair pathways such as non-homologous end joining (NHEJ) and alternative end joining (alt-EJ), generating complex genomic rearrangements and chromosomal abnormalities (71). Even in HRR-proficient tumors, defects in other DNA repair pathways—including replication fork protection, DNA damage response checkpoints, and mitotic fidelity—contribute to genomic instability (72). The resultant persistent DNA damage creates a state of chronic innate immune stimulation that may, paradoxically, both promote and constrain tumor progression.

WGD represents another prominent feature of ovarian cancer evolution (73), occurring in approximately 30-40% of HGSOC tumors (74). WGD generates tetraploid genomes with elevated rates of chromosomal mis-segregation (11). Recent work demonstrates that ongoing WGD promotes evolvability while simultaneously driving immune dysregulation (75, 76). Tetraploid cells activate cGAS-STING through micronuclei generation, and the resulting type I IFN response can promote tumor immunogenicity (77–79). However, chronic activation can drive immune exhaustion and immunotherapy resistance. The relationship between WGD, STING activation, and immune evasion represents a frontier area of investigation with direct therapeutic implications.

### 3.2 BRCA1/2 deficiency: a paradigm for STING-dependent immunogenicity

BRCA1 and BRCA2 occupy central positions in the DNA damage response network, their functions extending beyond HRR to encompass replication fork protection, DNA damage checkpoint activation, and transcriptional regulation (80). Cells deficient in BRCA1 or BRCA2 exhibit spontaneous DNA damage and activation of cGAS-STING signaling, even without exogenous genotoxic stress (81). This basal DNA damage is accompanied by a type I interferon transcriptional program—a finding that has profound implications for the immunobiology of hereditary ovarian cancers (21).

BRCA deficiency induce NF-κB-dependent type I interferon signaling in fallopian tube epithelial cells, the presumptive cell of origin for HGSOC (82). This effect is mediated, at least in part, through ATM-dependent DNA damage signaling and the accumulation of cytosolic DNA. Interestingly, the interferon response in BRCA1-deficient cells is not purely STING-dependent—alternative pathways involving MAVS and mitochondrial RNA sensing may also contribute—but STING plays a central role in amplifying and sustaining the response (83).

Beyond the direct effects on interferon signaling, BRCA1/2 deficiency shapes the tumor immune microenvironment in ways that are both beneficial and detrimental. The increased immunogenicity of BRCA-deficient tumors correlates with higher levels of tumor-infiltrating lymphocytes (TILs) and improved responses to immune checkpoint blockade (84–86). This has been attributed, in part, to the increased neoantigen burden resulting from genomic instability, but the role of STING-dependent innate immune signaling is increasingly appreciated as a key contributor. BRCA1-deficient ovarian cancers exhibit elevated expression of ISGs and chemokines that recruit T cells, consistent with STING activation (84). At the same time, chronic interferon signaling can induce expression of immune checkpoint molecules, including PD-L1, and promote the recruitment of immunosuppressive cell populations that limit antitumor immunity (87). Thus, BRCA deficiency creates an “immunogenic but restrained” phenotype that is potentially amenable to therapeutic manipulation.

The relationship between BRCA deficiency and STING signaling is further complicated by evidence that BRCA1 itself may directly regulate innate immune signaling independently of its role in DNA repair (43, 88). Recent studies have identified BRCA1 as a component of the type I interferon response, with BRCA1 deficiency leading to enhanced interferon signaling and ISG (81). The mechanisms underlying this effect include BRCA1’s ability to regulate NF-κB activity and its role in modulating chromatin accessibility at interferon-regulated genes. This direct regulatory function expands the potential for therapeutic intervention in BRCA-deficient ovarian cancers beyond the traditional focus on DNA repair pathways.

### 3.3 Beyond BRCA: other DDR defects that activate STING

While *BRCA1/2* mutations are the best-characterized DDR defects in ovarian cancer, other genetic alterations also converge on the cGAS-STING pathway. *CDK12* mutations, present in particularly of 3-5% of HGSOC, are associated with increased genomic instability and enhanced responsiveness to immune checkpoint blockade (89). The mechanistic basis for this immunogenicity involves CDK12’s role in maintaining transcriptional fidelity and genomic stability. CDK12 loss leads to DNA double-strand break accumulation and homologous recombination deficiency, which in turn activates the cGAS-STING pathway (90, 91). The resulting type I interferon response enhances tumor immunogenicity and promotes CD8^+^ T cell infiltration, potentially explaining the improved responses to immunotherapy observed in CDK12-mutant ovarian cancers.

*PPP2R1A* mutations occur in ovarian clear cell carcinoma and endometrial cancer and have been implicated in STING regulation (92). PP2A functions as a negative regulator of the cGAS-STING pathway, with PP2Ac deficiency enhancing STING signaling and antigen presentation (93). *PPP2R1A* mutations may therefore represent a genetic determinant of immune responsiveness in gynecologic cancers, offering a potential biomarker for patient selection and a rationale for therapeutic targeting of PP2A.

*SMARCA4* deficiency has similarly been linked to enhanced tumor immunogenicity (94). SMARCA4 encodes a catalytic subunit of the SWI/SNF chromatin remodeling complex, and its loss is associated with genome-wide chromatin accessibility alterations (95). SMARCA4-mutant ovarian cancers exhibit elevated type I interferon signaling and enhanced immune infiltration through STING-dependent mechanisms (94). SMARCA4 loss also impairs NK cell-mediated killing of senescent cells, promoting their accumulation and contributing to immune modulation (96).

The diversity of DDR defects that converge on STING activation highlights the centrality of this pathway in the immune biology of ovarian cancer (97). Identifying which DDR defects are most strongly associated with STING activation and immune sensitivity—and understanding how to therapeutically exploit this association—represents a key priority. The dual role of STING signaling in the ovarian cancer TME is illustrated in **Figure 2**.

**Figure 2:**
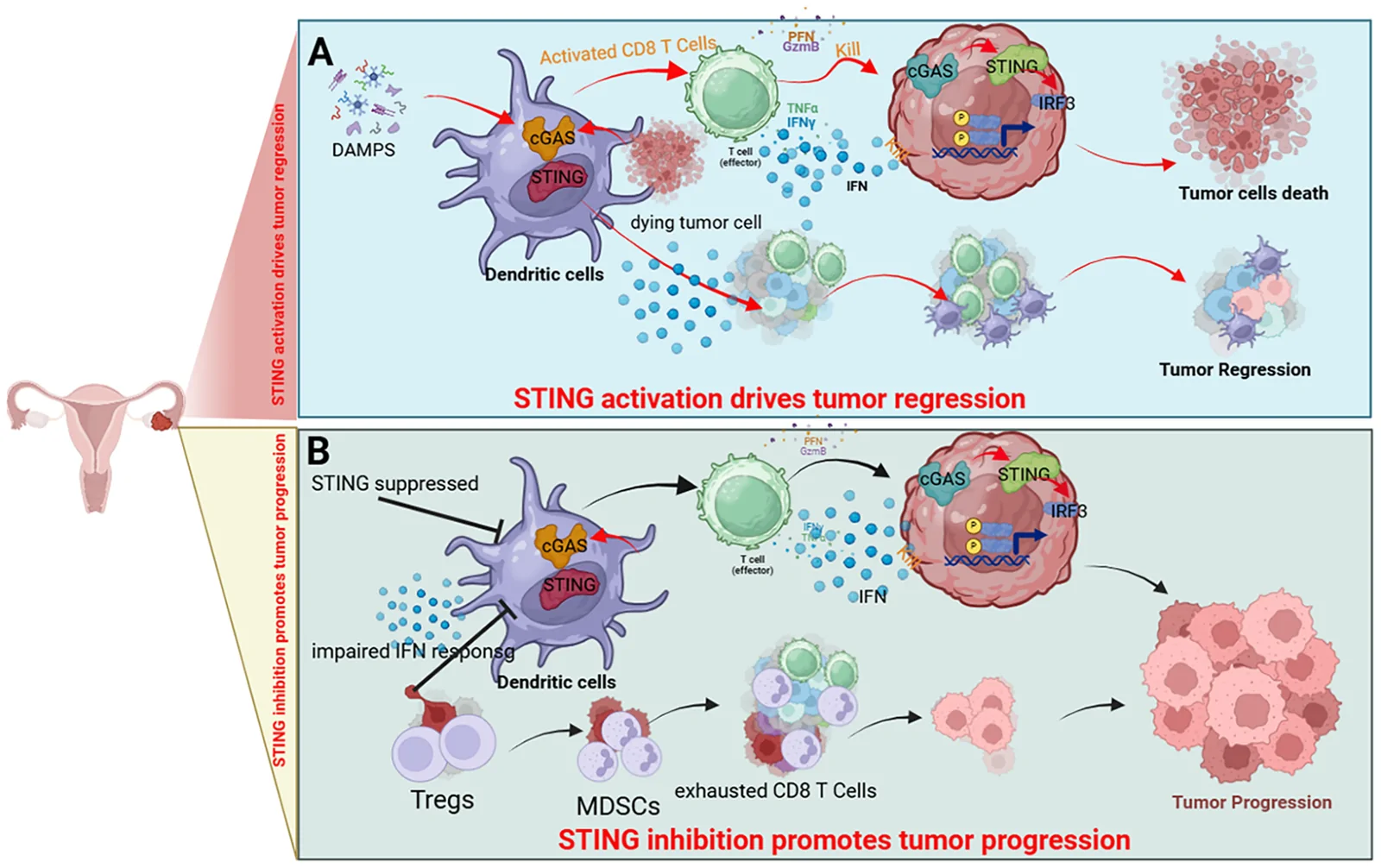
The dual role of STING signaling in the ovarian cancer TME. **(A)** Acute STING activation in tumor cells and DCs promotes DC maturation, antigen cross-presentation, and secretion of CXCL9 and CXCL10, recruiting CD8^+^ CTLs, and establishing antitumor immunity. **(B)** Under chronic STING stimulation or in specific genetic contexts (e.g., WGD), STING drives immunosuppression: (1) tumor cells upregulate PD-L1, causing CD8^+^ T-cell exhaustion; (2) paracrine WNT ligands activate β-catenin in CAFs, promoting POSTN^+^ myCAF differentiation; (3) POSTN^+^ myCAFs expand eTregs and MDSCs, driving fibrosis, immunosuppression, and PARPi resistance.

## 4 PARP inhibitors: bridging DNA repair and innate immunity

### 4.1 PARPi as immunomodulators: beyond synthetic lethality

PARP inhibitors have fundamentally altered the therapeutic landscape for BRCA-deficient ovarian cancers, yet their effects extend well beyond the cytotoxic synthetic lethality. PARPi exert profound immunomodulatory effects, with the cGAS-STING pathway serving as a key mediator of these effects (98, 99). PARP inhibition in BRCA-deficient cells leads to accumulation of DNA double-strand breaks and replication stress, generating cytosolic DNA fragments that activate cGAS and subsequently STING (6, 34, 99). This activation produces type I interferons and chemokines that promote T cell recruitment and activation.

PARPi-induced antitumor immunity is STING-dependent; STING-deficient tumors fail to mount effective immune responses following PARP inhibition. Pantelidou and colleagues demonstrated that CRISPR-mediated knockout of STING in cancer cells prevents proinflammatory signaling and is sufficient to abolish olaparib-induced T-cell infiltration *in vivo* (98). This finding has been corroborated in STING-deficient tumor models, where STING depletion reversed the antitumor effect of PARP inhibition (100). In ovarian cancer specifically, PARPi-triggered immune responses in BRCA1/2-mutated cells are mediated through cGAS/STING pathway activation, with STING signaling driving CD8^+^ T cell effector function (101, 102).

Beyond the initial activation of STING, PARPi induce a broader transcriptional program that enhances tumor immunogenicity. Upregulation of MHC class I molecules, increased expression of chemokines such as CXCL10 and CCL5, and enhanced cross-presentation of tumor antigens by DCs have all been described in PARPi-treated tumors (34, 103, 104). These changes collectively promote the infiltration of CD8^+^ T cells into the TME and enhance their effector function, creating a state of “immune-hot” inflammation that may overcome the relative immunotherapy resistance characteristic of ovarian cancer.

The magnitude of the PARPi-induced immune response depends on the specific PARP inhibitor, the tumor’s genetic background, and the TME state. Different PARP inhibitors exhibit distinct effects on STING signaling (105), and the underlying DDR deficiency influences immune response patterns. Understanding these differences is essential for optimizing combination regimens and patient selection. The immunomodulatory mechanisms of PARP inhibitors via the cGAS-STING axis in BRCA-deficient ovarian cancer are depicted in **Figure 3**.

**Figure 3:**
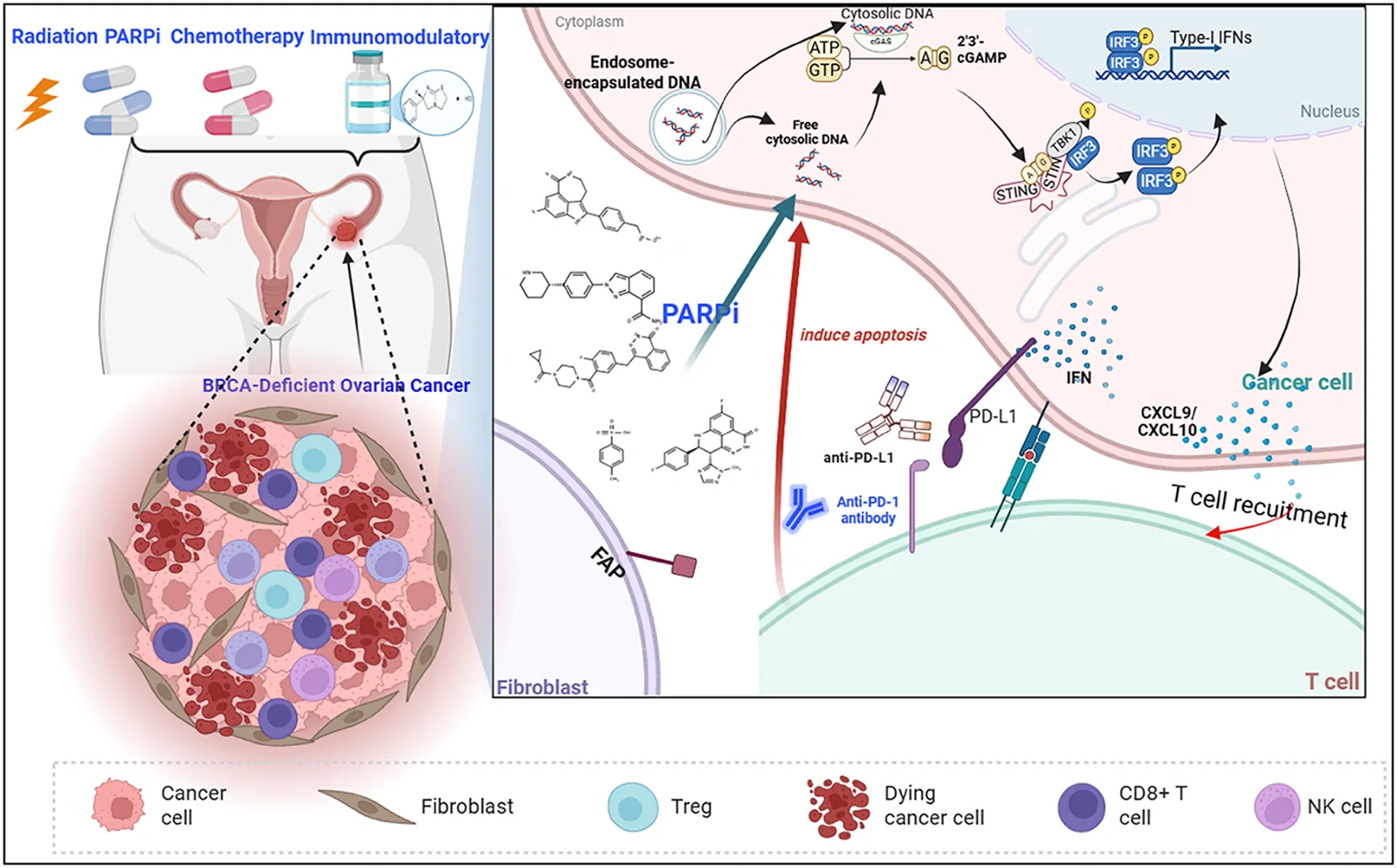
Immunomodulatory mechanisms of PARP inhibitors via the cGAS–STING axis in BRCA-deficient ovarian cancer. PARPi treatment in BRCA-deficient ovarian cancer induces persistent DNA damage and micronuclei formation, releasing cytosolic dsDNA that activates cGAS-STING. STING triggers TBK1-IRF3 signaling, inducing type I IFNs and chemokines (CXCL9/10) that recruit CD8^+^ T cells, supporting PARPi-ICB combinations. However, sustained STING activation upregulates tumor PD-L1 and drives CAF-mediated immunosuppression via the STING-WNT-POSTN axis, limiting durable PARPi efficacy and suggesting combination strategies targeting checkpoints or CAF pathways.

### 4.2 PARPi and immune checkpoint inhibition: rationale and evidence

The immunomodulatory effects of PARPi provide a compelling rationale for combining these agents with ICBs. PARPi-induced STING activation recruits T cells into the TME, while ICBs release the brakes on those T cells, enabling them to eliminate tumor cells. This combination has shown considerable promise in preclinical models, with PARPi plus anti-PD-1/PD-L1 or anti-CTLA-4 achieving enhanced antitumor efficacy compared with either agent alone (84, 86, 106).

Several clinical trials have evaluated the PARPi-ICB combination in ovarian cancer. In the initial phase II MEDIOLA study cohort of germline BRCA-mutated platinum-sensitive relapsed ovarian cancer (N = 32), olaparib plus durvalumab showed an ORR of 71.9% and a 28-week disease control rate (DCR) of 65.6% (12). Similarly, the KEYNOTE-162 trial evaluated pembrolizumab plus niraparib in patients with recurrent ovarian cancer, regardless of BRCA status, and observed encouraging response rates in BRCA-mutant tumors (107). More recent studies have extended these observations, with the COUPLET phase Ib trial demonstrating that rucaparib followed by combination rucaparib and atezolizumab was feasible and showed promising activity in advanced gynecologic and triple-negative breast cancers (13). These key clinical trials are summarized in **Table 1**.

**Table 1:** Key clinical trials of PARP inhibitors and immunotherapy combinations in ovarian cancer.

Trial	Combination	Patient population	Key outcomes	Clinical implications	Ref.
TOPACIO/KEYNOTE-162	Niraparib + Pembrolizumab	Recurrent ovarian cancer (N = 62), regardless of BRCA status	ORR 18% overall; 29% in BRCA-mutant; modest activity in BRCA wild-type	Benefit largely restricted to BRCA-mutant tumors; limited activity in unselected populations	(172)
MEDIOLA (Cohort 1)	Olaparib + Durvalumab	gBRCA-mutated platinum-sensitive relapsed ovarian cancer (N = 32)	ORR 71.9%; 28-week DCR 65.6%	Highly promising in biomarker-selected population; supports BRCA testing for patient selection	(12)
COUPLET (Phase Ib)	Rucaparib + Atezolizumab	Advanced gynecologic cancers and TNBC (N = 49)	Feasible; preliminary activity in BRCA-mutant tumors	Sequential rucaparib followed by combination may optimize STING activation and ICB response	(13)
JAVELIN Ovarian 100	Chemotherapy ± Avelumab → Avelumab maintenance	First-line ovarian cancer (N = 998), unselected	No PFS benefit: HR 1.14 (95% CI 0.83-1.56) for chemo+avelumab→avelumab; HR 1.43 (95% CI 1.05-1.95) for chemo→avelumab maintenance	Unselected patient populations unlikely to benefit; biomarker analyses (PD-L1, CD8, gBRCA) did not identify predictive subsets	(108)

However, the clinical results have been mixed, with some trials failing to show a clear benefit for the combination over PARPi alone. The JAVELIN Ovarian 100 trial, which evaluated avelumab in combination with or following platinum-based chemotherapy in first-line ovarian cancer, did not demonstrate improved progression-free survival with the addition of avelumab (108). Several explanations for these disappointing results have been proposed. First, the immunomodulatory effects of PARPi may be attenuated in the presence of an already immunosuppressive TME, particularly in tumors with high CAF infiltration or elevated PD-L1 expression. Second, the timing of ICB administration relative to PARPi may be critical: administering ICB before PARPi has primed the immune response may limit synergy. Third, the benefits of PARPi-ICB combinations appear to be largely restricted to BRCA-mutant populations, with BRCA wild-type tumors showing more modest responses. Fourth, the specific PARP inhibitor used may influence the magnitude of STING activation, as different PARP inhibitors exhibit distinct effects on innate immune signaling. These explanations are not mutually exclusive, and multiple factors likely contribute to the mixed clinical results observed to date.

### 4.3 Resistance to PARPi: the STING connection

PARPi resistance is a major clinical challenge, and recent work has identified STING signaling as a critical determinant, with the pathway playing a dual role: acute STING activation promotes antitumor immunity, whereas chronic signaling drives resistance through multiple mechanisms (10).

One mechanism involves the induction of immunosuppressive programs in the TME. STING activation in ovarian cancer cells drive the secretion of WNT ligands, particularly WNT3a and WNT7a, which act on CAFs to induce a protumorigenic phenotype characterized by production of periostin (POSTN) and other extracellular matrix proteins (44, 109). The POSTN^+^ CAF phenotype is associated with increased infiltration of regulatory T cells (eTregs) and exhausted CD8^+^ T cells, creating an immunosuppressive microenvironment that supports PARPi resistance (110, 111). Notably, POSTN blockade or WNT pathway inhibition reversed this resistance (10), suggesting that targeting the STING-WNT-POSTN axis could restore PARPi sensitivity.

A second mechanism involves the induction of immune checkpoint molecules. Chronic STING signaling upregulates PD-L1 on tumor cells, creating an adaptive resistance mechanism that limits the effectiveness of PARPi-induced T cell infiltration (112). In ovarian cancer, PARylation-mediated regulation of the cGAS/STING pathway has been implicated in PD-L1 upregulation and immune evasion (113). Mechanistically, this dualistic nature—whereby STING activation simultaneously promotes antitumor immunity and drives immune checkpoint upregulation—represents a key therapeutic challenge that has informed the rationale for combining PARP inhibitors with PD-1/PD-L1 blockade (114). Studies have further demonstrated that tumor-intrinsic PD-L1-mediated DDR inhibitor resistance is dependent on tumor STING signaling (115). This phenomenon explains, in part, the rationale for combining PARPi with PD-1/PD-L1 blockade—the PARPi recruits T cells, while the ICB relieves the PD-L1-mediated suppression that PARPi induces. Interestingly, the magnitude of PD-L1 upregulation appears to be modulated by the tumor’s genetic background, with BRCA1-deficient tumors showing variable PD-L1 induction that correlates with the strength of STING activation.

A third resistance mechanism involves the activity of the dipeptidyl peptidase 4 (DPP4). PARP inhibition induces a chemokine response that promotes T cell infiltration, but this response is limited by DPP4-mediated cleavage of chemokines such as CXCL10 (116, 117). DPP4 inhibition enhances the PARPi-induced chemokine response, increasing T cell infiltration and reducing tumor growth (118, 119). This finding suggests that targeting DPP4 could be a strategy for enhancing the immunomodulatory effects of PARPi and overcoming resistance.

Emerging evidence also implicates miR-181a in PARPi resistance through STING signaling. miR-181a targets STING mRNA, reducing its expression and thereby impairing the innate immune response to PARPi (120). Elevated miR-181a expression in PARPi-resistant ovarian cancer cells can be reversed by miR-181a inhibition, restoring STING expression and PARPi sensitivity.

### 4.4 Beyond PARPi: other DDR-targeting agents and STING

While PARP inhibitors have received the most attention in the context of ovarian cancer and STING activation, other DNA damage response-targeting agents also converge on this pathway and may offer complementary therapeutic opportunities. WEE1 inhibitors represent one promising class, with evidence that WEE1 inhibition activates the endogenous retrovirus (ERV) and double-stranded RNA pathway, inducing an interferon response that promotes antitumor immunity (121). WEE1 is a key regulator of the G2/M checkpoint, and its inhibition leads to premature mitotic entry and accumulation of DNA damage. The resulting genomic instability drives cytosolic DNA accumulation and STING activation, producing an immunogenic tumor phenotype (122). Notably, sequential PARPi followed by WEE1i produces robust immune responses in preclinical ovarian cancer models (123).

CHK1 inhibitors similarly activate STING through DNA damage accumulation. Prexasertib, a CHK1 inhibitor, has been evaluated in ovarian cancer clinical trials and has been shown to induce both innate and adaptive immune responses (124). In multiple tumor types, including preclinical models of ovarian cancer, CHK1 inhibition has been shown to potentiate antitumor immunity, an effect linked to the upregulation of MHC class I molecules, enhanced T cell infiltration, and improved sensitivity to ICB (125, 126). Ongoing clinical trials are evaluating CHK1 inhibitors in combination with ICB in ovarian cancer (124, 127).

The CDC7 inhibitor class has also emerged as a potential STING-activating strategy. CDC7 inhibition has been shown to potentiate the antitumor efficacy of PARPi in advanced ovarian cancer, with the combination producing enhanced STING-dependent immune responses (128). CDC7 inhibition leads to replication stress and DNA damage, activating the cGAS-STING pathway and promoting T cell infiltration. This approach may be particularly effective in tumors with defects in HRR or replication fork protection.

USP1 inhibition represents a newer strategy targeting a deubiquitinating enzyme involved in DNA repair. USP1 inhibition has been shown to enhance the efficacy of PARPi by disrupting HRR and promoting the accumulation of DNA damage (129). The resulting STING activation produces an immunogenic tumor phenotype that can be harnessed for combination immunotherapy (130–132). USP1 inhibitors are currently in early clinical development, and their potential to activate STING and enhance immunotherapy is an area of active investigation.

## 5 Tumor microenvironment: the STING nexus

### 5.1 Cancer-associated fibroblasts: amplifiers of STING-dependent immunosuppression

The ovarian cancer TME is a complex ecosystem in which cancer cells, stromal cells, and immune cells engage in dynamic interactions that shape disease progression and treatment response. Among the stromal components, CAFs have emerged as key players in mediating the immunological consequences of STING activation. The relationship between genomic instability in cancer cells and the functional state of CAFs exemplifies the intricate communication networks that operate within the TME.

The mechanism linking cancer cell genomic instability to CAF activation involves STING-dependent secretion of WNT ligands. Cancer cells with genomic instability activate STING, leading to the production of type I interferons and the transcriptional upregulation of WNT3a and WNT7a (133). These WNT ligands act in a paracrine manner on adjacent CAFs, activating the WNT/β-catenin pathway and inducing the expression of periostin (POSTN). In HGSOC, integrated single-cell transcriptomics and epigenomics have revealed that POSTN^+^ myofibroblast-like CAFs (myCAFs) are critical mediators of immunosuppression in tumors with high genomic instability (110, 111). Mechanistically, WNT/β-catenin signaling in fibroblasts establishes a POSTN-dependent positive feedback loop that epigenetically locks cells into the POSTN^+^ myCAF lineage. These myCAFs expand effector regulatory T cells (eTregs) and exhausted CD8^+^ T cells, converting immune activation into suppression and limiting PARPi efficacy. The immunosuppressive function of POSTN^+^ CAFs has also been observed in other malignancies, where they promote T-cell exclusion through reciprocal signaling with macrophages or drive M2 macrophage polarization (134, 135). In ovarian cancer specifically, therapeutic POSTN blockade reinvigorates T cell cytotoxicity and potentiates PARP inhibition, overcoming resistance (10). This pathway—the STING-WNT-POSTN axis—represents a critical point of communication between cancer cells and stroma that is therapeutically targetable.

The evidence for the STING-WNT-POSTN axis derives primarily from a landmark study by Liu and colleagues, which combined single-cell transcriptomics, epigenomics, and functional validation in mouse models. While these findings are mechanistically compelling and supported by human correlative data, several limitations should be acknowledged. The study was largely conducted in a single mouse model, and the generalizability to other ovarian cancer models and to the heterogeneity of human disease remains to be established. Moreover, the clinical correlative data, while suggestive, are not sufficient to establish causality in humans. Prospective validation in independent cohorts and, ultimately, in biomarker-stratified clinical trials will be required before this pathway can be translated into clinical practice.

The consequences of POSTN^+^ CAF accumulation are manifold. These CAFs promote the expansion of eTregs and the exhaustion of CD8^+^ T cells, creating an immunosuppressive microenvironment that suppresses antitumor immunity. This phenomenon has been directly demonstrated using a CAF conditional knockout system, in which POSTN-deficient CAFs fail to induce eTreg expansion or CD8+ T cell exhaustion (10). The immunosuppressive effects of POSTN+ CAFs are sufficient to cause resistance to PARP inhibitors, an effect that can be overcome by POSTN neutralization or WNT pathway inhibition (110, 111). Thus, therapeutic interventions that disrupt the STING-WNT-POSTN axis have the potential to restore PARPi sensitivity and enhance the effectiveness of immunotherapy.

The relevance of this pathway extends beyond PARPi resistance. In ovarian cancer patients with elevated POSTN expression, the POSTN^+^ CAF phenotype has been shown to correlate with increased eTreg infiltration, CD8^+^ T cell exhaustion, and poor prognosis (10). These findings suggest that POSTN is not merely a resistance mechanism but a driver of immune evasion more broadly. Strategies to inhibit POSTN or its upstream regulators may therefore have broader therapeutic applications in ovarian cancer beyond combination with PARP inhibitors.

Beyond the STING-WNT-POSTN axis, CAFs mediate STING-dependent immunosuppression through additional mechanisms. STING signaling in CAFs can directly promote the production of immunosuppressive cytokines including TGF-β and IL-10, which act to suppress T cell function (44, 136). Additionally, CAF-derived extracellular vesicles can carry STING signals to recipient cells, propagating immunosuppressive effects throughout the TME (137). Understanding these mechanisms and developing strategies to disrupt them represents an important direction for future investigation.

### 5.2 Myeloid cells: guardians, saboteurs, and dual agents

Myeloid cells—including tumor-associated macrophages (TAMs), DCs, and MDSCs—constitute a major fraction of the ovarian TME and are key responders to STING activation. The effects of STING on myeloid cells are context-dependent and sometimes paradoxical, reflecting the dualistic nature of the pathway that recurs throughout this review.

In DCs, STING activation is generally pro-immune. cGAS-STING signaling in DCs promotes their maturation, upregulates the expression of co-stimulatory molecules, and enhances the cross-presentation of tumor antigens (138–140). STING-activated DCs produce type I interferons and chemokines that recruit T cells, and they are essential for the initiation of T cell responses that culminate in antitumor immunity. This DC-mediated effect is the primary mechanism through which STING agonism has been shown to enhance immunotherapy responses in preclinical models.

In macrophages, the effects of STING are more nuanced. STING-activated macrophages can acquire an M1-like phenotype characterized by production of pro-inflammatory cytokines and enhanced phagocytic activity (141, 142). This M1 phenotype is associated with enhanced antitumor activity and recruitment of T cells. However, in the immunosuppressive TME of ovarian cancer, macrophages often acquire M2-like phenotypes that promote tumor growth and immune evasion (143). STING activation in M2 macrophages can, paradoxically, reinforce this immunosuppressive state through production of IL-10 and other immunosuppressive factors (104). The net effect of STING on macrophages therefore depends on the polarization state of the cells, which in turn reflects the cytokine milieu of the TME.

STING agonism has been shown to overcome resistance to PARPi in BRCA1-deficient breast cancer models through reprogramming of TAMs (142). The mechanism involves STING-mediated suppression of the immunosuppressive M2 phenotype and promotion of M1 polarization, leading to enhanced T cell infiltration and antitumor immunity. This macrophage reprogramming effect appears to be complementary to the direct effects of PARP inhibition on cancer cells, suggesting that combination strategies targeting both tumor cells and myeloid cells could be particularly effective.

Beyond macrophages, MDSCs represent a critical immunosuppressive population in ovarian cancer (144). MDSCs suppress T cell function through multiple mechanisms, including arginase activity, inducible nitric oxide synthase (iNOS), and production of immunosuppressive cytokines (145, 146). The effects of STING activation on MDSCs appear to be context-dependent, with some models showing reduced MDSC accumulation while others suggest chronic STING signaling may promote MDSC expansion, underscoring the complex role of this pathway in myeloid cell regulation. The relationship between STING and MDSCs is therefore context-dependent, and further investigation is needed to determine the optimal strategy for targeting these cells in ovarian cancer.

### 5.3 Spatial heterogeneity: different locations, different responses

Ovarian cancer is characterized by distinct metastatic sites—primary ovarian tumors, omental metastases, and peritoneal implants—each with unique microenvironmental properties that shape STING signaling and immune responses. Recent studies have revealed striking spatial heterogeneity in STING activation across these sites, with implications for both disease progression and therapeutic response.

Analysis of HGSOC samples has shown that the cGAS-STING pathway is activated to different extents in ovarian tumors compared with omental metastases (147). The dsDNA sensing/inflammation (DSI) score, a measure of cGAS-STING pathway activity, has been shown to be significantly higher in omental tumors compared with ovarian tumors, reflecting differences in genomic instability and innate immune signaling. This spatial heterogeneity in STING activation correlates with differences in immune infiltration and may contribute to the differential therapeutic responsiveness of tumors at different sites.

The distinct microenvironmental pressures exerted by different metastatic sites may explain these differences. Omental metastases encounter a microenvironment rich in adipose tissue and immune cells that may promote STING activation through multiple mechanisms, including metabolic stress and inflammatory signaling. Additionally, the distinct genomic evolution of metastases—which may acquire new mutations and chromosomal alterations during progression—can influence STING activation. The finding that STING activation varies spatially suggests that the choice of biopsy site may influence biomarker assessment and that systemic therapies targeting STING may produce site-specific effects.

Beyond spatial heterogeneity, temporal heterogeneity in STING activation is also apparent. STING activation in ovarian cancer changes over the course of disease progression and in response to therapy. Treatment with DNA-damaging agents such as platinum chemotherapy or PARPi increases STING activation, while the emergence of resistance may be accompanied by changes in STING signaling. Understanding these temporal dynamics is essential for optimizing the timing of STING-targeting therapies and assessing their effectiveness in patients.

## 6 Regulation of STING signaling: the molecular control panel

### 6.1 Negative regulators: the brakes on STING

The cGAS-STING pathway is subject to stringent negative regulation, reflecting the requirement to balance immune surveillance against the risks of excessive inflammation. In ovarian cancer, several negative regulators have been identified, and their perturbation may contribute to immune evasion and treatment resistance. Understanding these regulatory mechanisms has opened new avenues for therapeutic intervention, as targeting negative regulators can “release the brakes” on STING signaling and enhance antitumor immunity.

TRIM27 has emerged as a key negative regulator of the cGAS-STING pathway in ovarian cancer. TRIM27 is an E3 ubiquitin ligase that promotes the degradation of AMPK, a kinase that positively regulates STING activation (148). By destabilizing AMPK, TRIM27 suppresses cGAS-STING signaling and its downstream effects on interferon production and immune activation. The clinical relevance of this mechanism is supported by the observation that high TRIM27 expression in ovarian cancer patients correlates with poor prognosis and reduced immune infiltration. Targeting TRIM27 may therefore represent a strategy for enhancing STING signaling in ovarian cancer.

USP35, a deubiquitinating enzyme, functions as an additional negative regulator of STING signaling. USP35 removes ubiquitin chains from STING, preventing its degradation and prolonging signaling duration. While this might seem to enhance signaling, the net effect of USP35 is inhibitory, as STING signaling in the absence of USP35 is actually enhanced. The mechanism involves USP35-mediated stabilization of STING in a conformation that is less responsive to cGAMP binding, reducing its signaling capacity (149). USP35 overexpression in ovarian cancer has been associated with reduced STING activation and immune evasion, suggesting that USP35 inhibition could enhance STING-dependent antitumor immunity (149).

PPT1 (palmitoyl-protein thioesterase 1) is another negative regulator that has been identified in ovarian cancer. PPT1 removes palmitate groups from STING, reducing its stability and signaling activity (150). PPT1 inhibition has been shown to enhance STING signaling, promote T cell infiltration, and reverse the immunologically “cold” phenotype of ovarian tumors (150). The therapeutic potential of targeting PPT1 is supported by evidence that PPT1 inhibition synergizes with immune checkpoint blockade, enhancing antitumor immunity in preclinical models.

SETDB1-TRIM28 complex functions as a negative regulator of STING signaling through epigenetic mechanisms. The complex represses the expression of ISGs and other STING targets, limiting the immune response to STING activation (151). SETDB1 expression in ovarian cancer is associated with reduced immune infiltration and immunotherapy resistance (152), suggesting that targeting the SETDB1-TRIM28 complex could enhance STING-dependent antitumor immunity. Epigenetic modulators such as EZH2 inhibitors may have the capacity to reverse this repression and restore STING signaling.

N-MYC, an oncogenic transcription factor overexpressed in a subset of ovarian cancers, has been shown to impair innate immune signaling by suppressing the expression of cGAS and STING (153). N-MYC amplification in HGSOC is associated with reduced interferon signaling and immune infiltration, consistent with an immunosuppressive role. The relationship between N-MYC and STING suggests that targeting MYC-driven pathways could restore immune function in N-MYC-amplified tumors (153).

GRB2 (growth factor receptor-bound protein 2) represents yet another negative regulator of STING signaling, acting through its capacity to stabilize RAD51 at reversed replication forks (154). GRB2 binding to RAD51 promotes its stabilization and activity, suppressing the generation of cytosolic DNA and the resulting cGAS-STING activation. Mechanistically, GRB2 stabilizes RAD51 at stalled replication forks to prevent MRE11-mediated fork degradation, thereby limiting the accumulation of cytosolic DNA and subsequent cGAS-STING activation in ovarian cancer cells. Consequently, GRB2 overexpression in ovarian cancer is associated with reduced STING signaling and immune evasion. Importantly, GRB2 depletion in an ovarian cancer model activates cGAS-STING-dependent inflammatory responses and enhances the efficacy of PARPi therapy, suggesting that targeting GRB2 could enhance STING-dependent antitumor immunity (154).

### 6.2 Positive regulators and amplifiers

In contrast to the negative regulators that restrain STING signaling, several positive regulators amplify STING signaling and have been targeted for therapeutic enhancement. Understanding these positive regulators is essential for identifying strategies to boost STING-dependent antitumor immunity.

ZNFX1 has emerged as a master regulator of epigenetically induced pathogen mimicry and inflammasome signaling. ZNFX1 is a double-stranded RNA-binding protein that regulates the expression of endogenous retroviruses and other repetitive elements, promoting the accumulation of dsRNA and the activation of the cGAS-STING pathway (155). ZNFX1 deficiency leads to impaired STING signaling and reduced interferon responses, while ZNFX1 overexpression enhances STING activation and antitumor immunity (155). In ovarian cancer, ZNFX1 expression has been shown to correlate with immune infiltration and improved prognosis (155), suggesting that enhancing ZNFX1 could be a strategy for boosting STING signaling.

CENPM (centromere protein M) has been identified as a regulator of STING activation through its effects on pyroptosis. CENPM knockdown has been shown to activate the cGAS-STING pathway and promote pyroptosis in ovarian cancer cells (156). The enhanced STING signaling and pyroptosis resulting from CENPM knockdown are associated with increased antitumor immunity and reduced tumor growth (156). This finding suggests that targeting CENPM could be a strategy for inducing both STING activation and pyroptosis, generating a robust antitumor immune response.

Epigenetic regulators also function as positive modulators of STING activation. Loss of SMARCA4, as discussed earlier, enhances STING signaling through chromatin remodeling and increased accessibility at interferon-regulated genes. Similarly, CDK12 mutations promote STING activation and immune responsiveness (157). These findings suggest that certain genetic alterations in ovarian cancer confer increased STING signaling and represent opportunities for therapeutic exploitation. Indeed, the enhanced STING signaling that characterizes CDK12-mutant tumors may be the mechanistic basis for their favorable response to immunotherapy.

### 6.3 The role of metabolic regulation

Metabolic pathways intersect with STING signaling at multiple levels, adding another layer of regulatory complexity. In ovarian cancer, alterations in metabolism resulting from recurrent mutations and the unique environmental demands of peritoneal dissemination influence STING activation and immune responses.

Lactic acid accumulation, a hallmark of the ovarian cancer TME, has been shown to suppress STING signaling by inhibiting TBK1 activity (158). The acidic microenvironment created by glycolysis-driven lactate production impairs the phosphorylation of IRF3 and the production of type I interferons, reducing the immune response to STING activation (159). This metabolic suppression of STING signaling may contribute to the relative immunotherapy resistance of ovarian cancer and suggests that targeting tumor metabolism could enhance STING-dependent antitumor immunity.

NAD+ metabolism has been linked to STING regulation through the SURF4-STING axis, which has been shown to enhance T cell antitumor effects in the ovarian cancer microenvironment (109). This pathway involves NAD+-dependent deacetylation and regulation of SURF4, which modulates STING signaling and its effects on immune function. The findings suggest that targeting NAD+ metabolism could enhance STING signaling and T cell function in ovarian cancer.

Mitochondrial metabolism also intersects with STING signaling, as mitochondrial DNA release can activate cGAS and STING. Mitochondria are a source of cytosolic DNA under conditions of stress, including those induced by therapy. The release of mitochondrial DNA triggers cGAS-STING signaling and the production of type I interferons, contributing to the antitumor immune response (160). The regulation of mitochondrial DNA release—through mechanisms involving mitochondrial permeability transition and autophagy—therefore represents a potential point of therapeutic intervention.

## 7 Therapeutic strategies: from agonism to nanoparticle delivery

### 7.1 STING agonists: progress and challenges

Direct pharmacological activation of STING has been a longstanding goal in immuno-oncology, and several STING agonists have entered clinical development. The rationale for STING agonism is straightforward: activating the innate immune sensor induces type I interferon production and T cell activation, breaking immune tolerance and promoting antitumor immunity. However, the development of STING agonists suitable for clinical use has been challenging, reflecting the constraints of drug delivery and the dualistic nature of STING signaling. Therapeutic strategies, combinations, and future directions for targeting STING in ovarian cancer are summarized in **Figure 4**.

**Figure 4:**
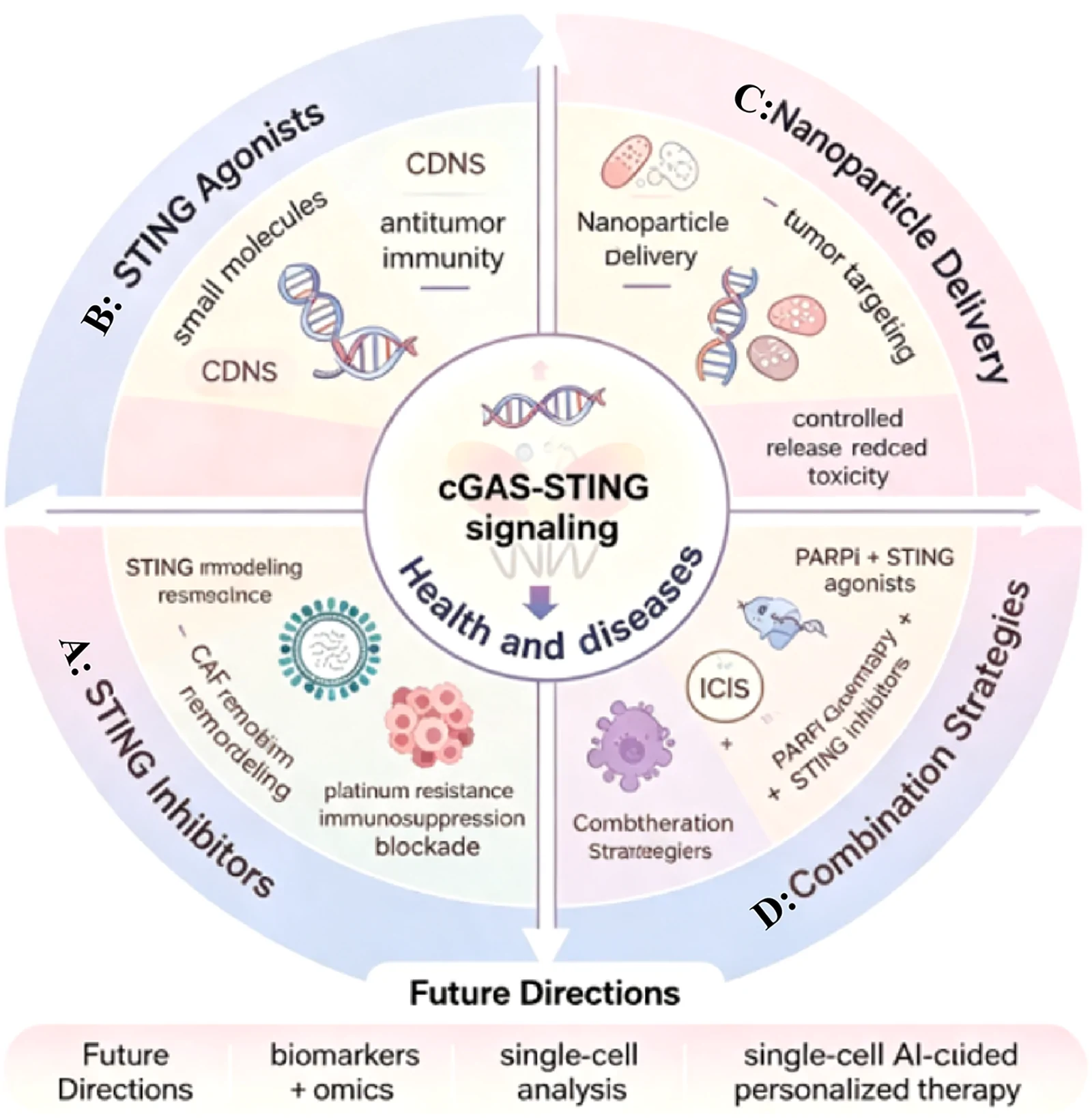
Therapeutic strategies, combinations, and future directions for targeting STING in ovarian cancer. **(A)** STING inhibitors to block CAF-mediated immunosuppression. **(B)** STING agonists (CDNs and non-nucleotide small molecules). **(C)** Nanoparticle delivery systems for tumor-targeted delivery. **(D)** Rational combinations: PARPi + STING agonists/ICB to overcome PARPi resistance; STING agonists + ICB to synergistically boost T-cell priming; chemotherapy + STING inhibitors to abrogate CAF-mediated immune exclusion. Core challenges include predictive biomarkers, resistance mechanisms, and toxicity management. Future research will leverage spatial omics, single-cell analysis, and AI to enable personalized therapeutic schedules.

First-generation STING agonists, including cyclic dinucleotides (CDNs) such as cGAMP and synthetic analogs, have demonstrated potent antitumor activity in preclinical models (161, 162). However, their clinical development has been hampered by poor pharmacokinetics, including rapid degradation and limited tumor penetration. Intratumoral administration has been the primary delivery route, limiting their application to accessible tumors. Systemic administration of CDNs has been associated with immune-related toxicities and limited efficacy, reflecting the systemic inflammatory response that can result from widespread STING activation (163).

Non-nucleotide STING agonists, including small molecule agonists developed through high-throughput screening, have offered advantages in terms of pharmacokinetics and oral bioavailability (164). However, these molecules have also faced challenges, including limited tumor accumulation and systemic toxicities. The development of tumor-targeted STING agonists that achieve high concentrations in the TME while minimizing systemic exposure represents a priority for the field.

A critical analysis of the STING agonist literature reveals a significant disconnect between preclinical promise and clinical reality. While numerous murine studies have demonstrated robust antitumor efficacy with STING agonists, clinical trials have reported modest responses at best. This translational gap reflects multiple factors. First, first-generation STING agonists suffer from poor pharmacokinetics, requiring intratumoral administration and exhibiting limited tumor penetration. Second, the immunologically “hot” murine models commonly used in preclinical studies do not recapitulate the immunosuppressive TME of human ovarian cancer. Third, the potential for chronic STING activation to drive immunosuppression rather than immunity—a possibility that has not been adequately addressed in clinical trial design—may limit the efficacy of repeated dosing schedules. Fourth, the absence of validated biomarkers has prevented the identification of patients most likely to respond, leading to the enrollment of unselected populations. These four barriers are not insurmountable, but overcoming them will require a coordinated effort across drug development, preclinical modeling, and clinical trial design.

STING agonist combinations have been explored to enhance efficacy and overcome limitations. STING agonists combined with PARP inhibitors have shown particular promise, with preclinical evidence that the combination enhances antitumor immunity and overcomes PARPi resistance (142). STING agonists combined with ICB have also been evaluated, building on the premise that STING activation recruits T cells while ICB maintain their effector function. Clinical trials evaluating STING agonists in combination with ICB and DDR-targeting agents are ongoing, and the results will be critical for defining the future role of STING agonism in ovarian cancer therapy.

### 7.2 Nanoparticle delivery systems: precision STING activation

Nanoparticle delivery platforms have emerged as transformative tools for STING-based therapies, offering the potential for enhanced tumor targeting, controlled release, and reduced systemic toxicity. The design of effective nanoparticle STING delivery systems requires careful consideration of multiple parameters, including nanoparticle composition, size, and surface chemistry, as well as the specific STING agonist incorporated.

Metal-phenolic network nanoparticles represent one class of STING-targeting nanoplatforms that has been evaluated in ovarian cancer (165). These particles self-assemble from polyphenols and metal ions, enabling the incorporation of STING agonists and other therapeutic payloads. The acidic TME promotes their dissolution and release of payloads, ensuring that STING activation is largely confined to the TME. In ovarian cancer models, metal-phenolic nanoparticles loaded with STING agonists have been shown to enhance antitumor immunity and sensitize tumors to PARP inhibition (140).

Polymer-based nanoparticles have also been developed for STING agonist delivery. These particles protect their payloads from degradation, enable extended circulation and tumor accumulation, and allow for the co-delivery of multiple agents that enhance STING signaling (166). The development of “single-molecule multitarget” nanoparticles that co-deliver STING agonists with inhibitors of STING-negative regulators represents an advance in this area (167). Such particles enable simultaneous activation of STING and inhibition of its feedback regulators, potentiating the STING response while limiting adaptive resistance.

Thermosensitive gels have been developed for localized STING delivery in ovarian cancer (168). These formulations are liquid at room temperature and gel at body temperature, allowing for injection into the peritoneal cavity and retention at tumor sites. The co-delivery of STING agonists, chemotherapeutics, and CAF inhibitors in a single thermosensitive gel represents a strategy for modulating multiple aspects of the TME (169). This approach has demonstrated enhanced antitumor immunity and reduced tumor growth in ovarian cancer models.

Targeted nanoparticles carrying STING agonists and other payloads exploit tumor-specific receptors for enhanced uptake and efficacy. Folic acid-modified nanoparticles have been developed for ovarian cancer targeting, taking advantage of the high expression of folate receptors on ovarian cancer cells (170). These particles can serve as *in situ* tumor vaccines, inducing a robust antitumor immune response and preventing metastasis and recurrence.

### 7.3 STING inhibition: the rationale for a paradoxical approach

While STING activation is generally considered a strategy for enhancing antitumor immunity, there is growing recognition that STING inhibition may also have therapeutic value, particularly in the context of platinum chemotherapy. This paradoxical approach reflects the dualistic role of STING in the TME—while acute STING activation promotes immunity, chronic STING activation can drive immunosuppression and resistance.

The evidence for STING inhibition in ovarian cancer comes from studies showing that STING signaling in CAFs drives immunosuppression and platinum chemotherapy resistance. Jiale Liu and colleagues demonstrated that CAF-specific STING signaling promotes the secretion of immunosuppressive factors that inhibit T cell function and confer resistance to platinum-based chemotherapy (58). STING inhibitors have been shown to sensitize ovarian cancer cells to platinum chemotherapy *in vivo*, an effect that was dependent on CAF STING signaling (58). These findings suggest that STING inhibition could be a valuable strategy in combination with platinum-based chemotherapy, particularly in patients with CAF-rich tumors.

The rationale for STING inhibition extends to PARPi resistance as well. Chronic STING signaling has been shown to drive PARPi resistance through induction of immunosuppressive programs, as discussed earlier. STING inhibitors may therefore have utility in overcoming PARPi resistance, either by directly inhibiting STING or by targeting its downstream effects. The clinical application of STING inhibitors would require careful patient selection, as not all patients would benefit from STING inhibition, and STING activation may be the preferred strategy in tumors with intact immunity.

The development of STING inhibitors is still in early stages, but several small molecule inhibitors have been identified and are being evaluated in preclinical models. The clinical development of STING inhibitors for cancer therapy will require careful consideration of patient selection, biomarkers, and combination with other agents. Understanding which tumors are most likely to benefit from STING inhibition—and which from STING activation—represents a key area for future investigation.

## 8 Clinical perspectives and translational challenges

### 8.1 Biomarkers of STING activation and sensitivity

The clinical success of STING-targeting therapies in ovarian cancer will depend, at least in part, on the ability to select patients most likely to benefit. Biomarkers of STING activation and sensitivity are needed to guide patient stratification and monitor therapeutic efficacy.

#### 8.1.1 Tier 1: clinically validated biomarkers

BRCA1/2 and HRD status represent the most clinically validated biomarkers related to STING-dependent immunogenicity (68–70). BRCA1/2 mutations are established predictors of PARPi response and have been associated with enhanced immune infiltration. HRD status, assessed through genomic scar assays, is similarly validated for PARPi selection. These biomarkers are already used in clinical practice and can guide patient selection for PARPi-ICB combinations.

#### 8.1.2 Tier 2: promising translational biomarkers

STING pathway activity signatures, including the DSI score, reflect functional pathway activity rather than just protein expression (147). The DSI score correlates with type I interferon signaling and immune infiltration in ovarian cancer and may predict response to STING-targeting therapies. However, prospective clinical validation is required (147). CDK12 mutation status has been associated with enhanced STING activation and immunogenicity (89–91), though the prevalence in ovarian cancer is low (3-5%).

#### 8.1.3 Tier 3: experimental biomarkers requiring validation

POSTN-positive CAF signatures have demonstrated mechanistic relevance in preclinical models and correlative clinical studies (10, 171), but require prospective validation. POSTN expression as measured by immunohistochemistry or transcriptomics shows promise as a biomarker of resistance and response. Immune infiltration patterns, including CD8^+^ T-cell density and Treg/CD8 ratio, reflect TME status and may influence therapeutic response, but their role as biomarkers for STING-targeting therapies is not established. STING protein expression by immunohistochemistry, while technically feasible, does not capture the functional state of the pathway and has shown variable correlation with outcomes (171).

The path from biomarker discovery to clinical implementation follows a well-defined, sequential process. It begins with technical validation and assay standardization, ensuring that candidate biomarkers can be measured reliably and reproducibly across different laboratories and platforms. This foundational step is followed by retrospective validation in independent cohort studies, which tests whether the biomarker retains its predictive or prognostic value in diverse patient populations. The third and most rigorous step is prospective validation in biomarker-enriched clinical trials, where biomarker-guided patient selection is tested against standard-of-care approaches in a controlled, hypothesis-driven manner. The final, definitive step is demonstration of clinical utility, showing that biomarker-guided management genuinely improves patient outcomes—whether through enhanced response rates, prolonged survival, or reduced toxicity—in a manner that justifies its integration into routine clinical practice. In the context of ovarian cancer, most candidate biomarkers for STING-targeting therapies currently remain at the first two stages, with prospective validation and clinical utility demonstration representing the critical next frontier.

### 8.2 Clinical trial landscape: lessons from successes and failures

Multiple clinical trials have evaluated PARP inhibitor-immune checkpoint blockade combinations and STING-targeting therapies in ovarian cancer. The results have been mixed, and understanding why some strategies have succeeded while others have failed provides critical insights for future development.

#### 8.2.1 Critical analysis of translational failures

The modest efficacy of STING agonists in early clinical trials—and the failure of several PARPi-ICB combinations to demonstrate benefit in unselected populations—reflects multiple barriers to clinical translation.

First, preclinical models have consistently failed to predict clinical outcomes. Most murine studies use immunologically “hot” syngeneic models that do not recapitulate the immunosuppressive TME of human ovarian cancer. Humanized models and patient-derived xenografts may offer incremental improvements, but even these fall short. Second, achieving adequate tumor concentrations of STING agonists without systemic toxicity has proven challenging. First-generation cyclic dinucleotides require intratumoral injection and have limited tumor penetration, while systemic administration is associated with immune-related toxicities that may outweigh therapeutic benefits (163). Third, the dualistic nature of STING signaling—whereby chronic activation drives immunosuppression—means that dosing schedules must be carefully optimized. Most preclinical studies used acute, intermittent STING activation, while clinical protocols often involve repeated dosing that may promote immune tolerance or exhaustion. Fourth, the absence of validated predictive biomarkers has prevented patient selection, meaning that many trials included patients whose tumors lack the molecular features required for STING-dependent immunogenicity.

The lessons from these failures are clear. Future trial design must be guided by three core principles. First, patient selection should be driven by biomarker-based enrichment, targeting individuals with demonstrable baseline STING pathway activity or specific DNA damage repair (DDR) defects. This approach moves beyond the “one-size-fits-all” strategy that has proven ineffective in unselected populations. Second, dosing schedules must be carefully optimized to achieve acute, transient STING activation rather than chronic signaling, which is increasingly recognized as a driver of immunosuppression rather than antitumor immunity. This temporal dimension of STING modulation has been largely overlooked in early-phase trials but is likely critical for therapeutic success. Third, rational combinations must be designed to address the specific resistance mechanisms that are operative in ovarian cancer, including CAF-mediated immunosuppression, PD-L1 upregulation, and other adaptive immune evasion strategies. These three principles are not merely theoretical recommendations—they represent concrete, testable strategies that should be incorporated into the next generation of clinical trials for STING-targeting therapies.

### 8.3 Overcoming resistance to STING-based therapies

Resistance to STING-targeting therapies is a likely challenge, and understanding and overcoming this resistance is essential for effective clinical application. The mechanisms of resistance are likely diverse, reflecting the multiple regulatory pathways and TME influences on STING signaling.

Upregulation of negative regulators may limit the efficacy of STING agonists. Overexpression of negative regulators such as TRIM27, USP35, and PPT1 may reduce STING signaling and promote resistance (148–150). Therapeutic strategies that inhibit these negative regulators could restore STING signaling and overcome resistance. Small molecule inhibitors targeting these negative regulators are being developed, and their combination with STING agonists may prove synergistic.

Loss of downstream signaling components may also drive resistance. Mutations or epigenetic silencing of IRF3, TBK1, or other components of the STING signaling cascade could limit the response to STING agonists (42, 46). Tumors with such defects may be insensitive to STING activation, and alternative strategies may be needed for these patients.

Adaptive immune responses may also contribute to resistance. Tumors may upregulate PD-L1 and other immune checkpoints in response to STING activation, limiting the effectiveness of STING agonists. Combination with ICB may overcome this adaptive resistance, as discussed earlier.

Tumor evolution may lead to resistance through selection of clones with reduced STING signaling or increased immunosuppressive capacity. The development of resistance mechanisms over time suggests that dynamic monitoring of STING signaling may be needed to guide therapy, and that sequential combinations of agents may be necessary to maintain efficacy.

## 9 Future directions and concluding remarks

### 9.1 Unanswered questions

Despite the considerable progress summarized in this review, numerous questions about the role of cGAS-STING in ovarian cancer remain unanswered. These questions define the frontier of the field and will guide future investigation.

First, the mechanistic basis for the dualistic effects of STING signaling—its capacity to promote both immunity and immunosuppression—requires further elucidation. What are the determinants of whether STING activation leads to T cell activation or immune evasion? How do duration, intensity, and cell type-specific signaling contribute to these divergent outcomes? Understanding these determinants may reveal strategies for selectively promoting the immunogenic arm of STING signaling.

Second, the relationship between STING and other innate immune pathways in ovarian cancer requires further investigation. How does STING interact with other cytosolic DNA sensors and with sensors of RNA, such as the RIG-I-MAVS pathway? What is the contribution of the inflammasome and pyroptosis to STING-dependent immunity? Understanding these interactions may reveal new therapeutic opportunities.

Third, the optimal therapeutic strategies for targeting STING in ovarian cancer require definition. Which patients should receive STING agonists, and which should receive STING inhibitors? What are the optimal combination partners for each approach? What is the optimal schedule and duration of treatment? Clinical studies are needed to address these questions.

Fourth, the development of predictive biomarkers for STING-targeting therapies remains a priority. Which biomarkers will most effectively predict response to STING agonists and inhibitors? How can these biomarkers be integrated into clinical trial design and patient care? The development of a robust biomarker platform is essential for the clinical success of STING-targeting therapies.

### 9.2 Future framework and roadmap: from discovery to clinical translation

While the preceding section identified key unresolved questions, the field now requires a conceptual roadmap to guide the translation of mechanistic insights into clinical benefit. We propose the following integrated framework:

**Pillar 1: Spatial and Single-Cell Omics to Resolve Heterogeneity**.

The spatial and temporal heterogeneity of STING activation across ovarian cancer metastatic sites—as revealed by recent single-cell and spatial transcriptomic studies (11, 21)—demands a more granular understanding of STING biology.

Future investigations should pursue three complementary objectives. First, STING pathway activity should be characterized at single-cell resolution across the full spectrum of disease sites, encompassing primary ovarian tumors, omental metastases, and peritoneal implants. This spatial mapping is essential because the ovarian cancer TME is not a uniform entity; rather, it comprises distinct anatomical compartments, each with unique microenvironmental pressures that shape STING signaling and immune responses. Second, cell-type-specific signatures of STING activation must be identified to distinguish those that predict therapeutic response from those that signal resistance. Given that STING activation elicits divergent outcomes in cancer cells, CAFs, dendritic cells, and macrophages, resolving this cell-type specificity is critical for understanding both the immunostimulatory and immunosuppressive consequences of pathway engagement. Third, the evolution of STING signaling over the course of treatment should be tracked longitudinally to elucidate how resistance emerges and whether it is driven by intrinsic tumor adaptation, extrinsic microenvironmental changes, or both. These single-cell and spatial approaches should be integrated with spatial metabolomics to link STING activation to metabolic states, thereby capturing the functional consequences of pathway engagement in their native tissue context.

Pillar 2: Biomarker-Guided Patient Stratification.

The mixed results of clinical trials to date underscore the critical need for predictive biomarkers. The mixed results of clinical trials to date underscore the critical need for predictive biomarkers to guide patient selection and therapeutic decision-making. We propose a sequential, three-step approach to implementing biomarker-driven stratification in clinical practice and trial design. The first step is the development and analytical validation of multiplex assays capable of simultaneously capturing STING pathway activity, DDR status, and TME composition from routine clinical samples, such as tumor biopsies or, where feasible, liquid biopsies. Such assays must be robust, cost-effective, and compatible with existing clinical pathology workflows to facilitate widespread adoption. The second step involves the stratification of patients into molecularly defined subtypes based on these integrated assays. This stratification moves beyond single-marker approaches to recognize that response to STING-targeting therapies is likely determined by the convergence of multiple biological features—including the intrinsic immunogenicity of the tumor, the integrity of its DNA repair pathways, and the composition of its stromal and immune microenvironment. The third step is the assignment of patients to subtype-specific therapeutic strategies, thereby moving from a “one-size-fits-all” paradigm to a precision medicine framework. For example, patients with “STING-high, CAF-low” tumors—characterized by intrinsic pathway activation but limited stromal immune exclusion—might be optimal candidates for STING agonists combined with immune checkpoint blockade. Conversely, patients with “STING-high, CAF-high” tumors, where cancer-associated fibroblasts actively suppress T-cell infiltration and function, may require the addition of CAF-targeting agents to overcome this stromal barrier before STING modulation can exert its full therapeutic effect. This sequential framework is not prescriptive but rather illustrative of the principles that should guide biomarker-driven clinical development.

Pillar 3: Temporal Modulation of STING Activity.

The dualistic nature of STING signaling suggests that therapeutic success will depend on achieving acute rather than chronic activation (28, 29). We propose the following principles: (i) STING agonists should be administered in a pulsed schedule to achieve peak activation followed by signal termination; (ii) the duration of STING activation should be controlled through pharmacokinetic design and dosing optimization; and (iii) strategies that limit the duration of STING signaling—such as rapidly cleared agonists or inhibitors of STING degradation—may be preferable to sustained activation.

Pillar 4: Rational Sequencing of Therapeutic Agents.

Building on the principles outlined above, we propose a conceptual framework for the optimal sequencing of therapeutic agents to maximize the immunostimulatory potential of the cGAS-STING pathway while minimizing adaptive resistance. This framework is grounded in the biological premise that the timing of interventions can be as critical as their selection. First, PARP inhibitors should be administered as the initial priming agent in BRCA-deficient or HRD-positive tumors, where they induce persistent DNA damage, replication stress, and the accumulation of cytosolic DNA—the very substrate required for cGAS-STING activation. This PARPi-driven priming generates an “immune-hot” microenvironment characterized by STING-dependent type I interferon production, chemokine secretion, and subsequent CD8^+^ T-cell infiltration into the tumor. Second, following this PARPi priming phase—which typically requires several days to weeks to achieve maximal pathway engagement—immune checkpoint blockade should be introduced to prevent the premature exhaustion of recruited T cells and to maintain their effector function. The sequential rather than concurrent administration of these agents is conceptually important; PARPi-induced STING activation simultaneously upregulates PD-L1 as a feedback mechanism, and ICB is most effective when deployed precisely at the moment this adaptive resistance pathway is engaged. Third, STING agonists may be deployed as a “boost” at the time of PARPi initiation to amplify the initial innate immune response, or alternatively, as a salvage strategy in patients who show evidence of STING suppression—such as low baseline type I interferon signatures—and who may not mount an adequate response to PARPi alone. This flexible, temporally optimized approach recognizes that the biological effects of each agent are not static but evolve over time, and that careful sequencing may be required to achieve synergistic rather than merely additive effects. Importantly, this framework is hypothesis-generating rather than prescriptive; its clinical validity will need to be tested in biomarker-enriched, adaptive trial designs that incorporate longitudinal pharmacodynamic assessments.

This roadmap is not a rigid prescription but a flexible framework that will need to be iteratively refined as new data emerge. The guiding principle is that clinical success will require integration of mechanistic understanding, patient selection, and therapeutic timing—a precision approach that moves beyond “one-size-fits-all” strategies.

### 9.3 Concluding remarks

The cGAS-STING pathway has emerged as a central node in the immune biology of ovarian cancer, linking the genomic instability that defines this disease to innate immune surveillance and adaptive antitumor immunity. The pathway is at once a source of immunogenicity and a driver of immunosuppression, reflecting the dualistic nature of STING signaling that has been a recurring theme of this review.

What distinguishes this review from prior work is its integration of four critical perspectives. First, we have systematically examined the stromal response to STING activation, particularly the STING-WNT-POSTN axis in cancer-associated fibroblasts, as a mechanism that can subvert antitumor immunity and drive PARP inhibitor resistance. Second, we have highlighted spatial heterogeneity in STING activation across ovarian cancer metastatic sites, recognizing that the site of biopsy may influence biomarker assessment and that systemic therapies targeting STING may produce site-specific effects. Third, we have critically analyzed the clinical trial landscape, extracting lessons from negative studies and proposing specific strategies to overcome translational barriers. Fourth, we have proposed a conceptual roadmap that integrates biomarker-guided patient stratification, temporal modulation of STING activity, and rational sequencing of therapeutic agents.

The therapeutic implications of these insights are substantial. STING-targeting therapies—whether agonists, inhibitors, or modulators of its regulatory network—offer the potential to enhance antitumor immunity, overcome resistance to PARPi and chemotherapy, and improve outcomes for patients with ovarian cancer. The evidence for these strategies is strongest for the combination of PARPi with STING agonists, with preclinical studies showing robust synergy and clinical trials providing encouraging signals. The combination of STING-targeting therapies with ICB and other immunomodulatory strategies is also being actively evaluated.

Yet substantial challenges remain. The complexity of STING signaling—its contextual determinants, its cell-type specificity, and its dualistic outcomes—demands careful patient selection and therapeutic design. The development of predictive biomarkers and the integration of emerging technologies such as spatial omics and artificial intelligence are essential for the clinical success of STING-targeting therapies. The barriers to STING-targeting therapies—delivery, toxicity, and resistance—must be addressed through continued innovation in drug design and combination strategies.

As our understanding of the cGAS-STING pathway in ovarian cancer deepens, it is increasingly apparent that we are only beginning to uncover the therapeutic potential of this pathway. The convergence of insights from DNA repair biology, innate immunity, and tumor immunology has created an unprecedented opportunity to develop new therapeutic strategies for one of the most challenging of all malignancies. By building on the foundation of knowledge reviewed here, and by addressing the challenges that remain, we can realize the promise of STING-targeting therapies for patients with ovarian cancer. The path ahead is long, but the potential rewards are extraordinary….

## References

[1] Ray-Coquard I Wilson C Moore K . The Lancet Commission on ovarian cancer: towards equity-driven reform. Lancet. (2026) 407:743–4. doi: 10.1016/s0140-6736(25)02463-8 41520675

[2] Balkwill FR Laumont CM Burdett N Le Saux O Garsed DW Grither WR . Rethinking ovarian cancer III: the past decade and future directions. Nat Rev Cancer. (2026) 26:452–71. doi: 10.1038/s41568-026-00916-0 41975230

[3] Caruso G Weroha SJ Cliby W . Ovarian cancer: a review. JAMA. (2025) 334:1278–91. doi: 10.1001/jama.2025.9495 40690248

[4] Siegel RL Kratzer TB Wagle NS Sung H Jemal A . Cancer statistics, 2026. CA Cancer J Clin. (2026) 76:e70043. doi: 10.3322/caac.70043 41528114 PMC12798275

[5] Santoro A Angelico G Travaglino A Inzani F Spadola S Pettinato A . The multiple facets of ovarian high grade serous carcinoma: a review on morphological, immunohistochemical and molecular features. Crit Rev Oncology/Hematology. (2025) 208:104603. doi: 10.1016/j.critrevonc.2024.104603 39732305

[6] Zhang D Zhang B . cGAS/STING signaling pathway in gynecological malignancies: from molecular mechanisms to therapeutic values. Front Immunol. (2025) 16:1525736. doi: 10.3389/fimmu.2025.1525736 39949780 PMC11821648

[7] Li R Liu H Liu Y . The cGAS-STING pathway and female reproductive system diseases. Front Immunol. (2024) 15:1447719. doi: 10.3389/fimmu.2024.1447719 39445027 PMC11496054

[8] Singhai H Rehman U Bukhary DM Gupta G Patil UK Sahebkar A . STING-activating nanomedicines in cancer immunotherapy: mechanisms, design, and therapeutic outcomes. Int Immunopharmacol. (2026) 180:116692. doi: 10.1016/j.intimp.2026.116692 42035546

[9] Carlsen L El-Deiry WS . Anti-cancer immune responses to DNA damage response inhibitors: molecular mechanisms and progress toward clinical translation. Front Oncol. (2022) 12:998388. doi: 10.3389/fonc.2022.998388 36276148 PMC9583871

[10] Liu D Tao K Cai C Lin Y Yu R Xiong K . Genomic instability drives POSTN(+) myofibroblasts via STING-WNT axis to promote immunosuppression and PARPi resistance in ovarian cancer. Sci Transl Med. (2026) 18:eady2719. doi: 10.1126/scitranslmed.ady2719 42202048

[11] McPherson A Vázquez-García I Myers MA Al-Rawi DH Zatzman M Weiner AC . Ongoing genome doubling shapes evolvability and immunity in ovarian cancer. Nature. (2025) 644:1078–87. doi: 10.1038/s41586-025-09240-3 40670783 PMC12390843

[12] Drew Y Kim JW Penson RT O'Malley DM Parkinson C Roxburgh P . Olaparib plus durvalumab, with or without bevacizumab, as treatment in PARP inhibitor-naïve platinum-sensitive relapsed ovarian cancer: a phase II multi-cohort study. Clin Cancer Res. (2024) 30:50–62. doi: 10.1158/1078-0432.ccr-23-2249 37939124 PMC10767301

[13] Kristeleit R Leary A Oaknin A Redondo A George A Chui S . PARP inhibition with rucaparib alone followed by combination with atezolizumab: phase Ib COUPLET clinical study in advanced gynaecological and triple-negative breast cancers. Br J Cancer. (2024) 131:820–31. doi: 10.1038/s41416-024-02776-7 38971950 PMC11369183

[14] Li J Hubisz MJ Earlie EM Duran MA Hong C Varela AA . Non-cell-autonomous cancer progression from chromosomal instability. Nature. (2023) 620:1080–8. doi: 10.1038/s41586-023-06464-z 37612508 PMC10468402

[15] Farmer H McCabe N Lord CJ Tutt AN Johnson DA Richardson TB . Targeting the DNA repair defect in BRCA mutant cells as a therapeutic strategy. Nature. (2005) 434:917–21. doi: 10.1038/nature03445 15829967

[16] Lorusso D Xiang Y Hasegawa K Scambia G Leiva M Ramos-Elias P . Pembrolizumab or placebo with chemoradiotherapy followed by pembrolizumab or placebo for newly diagnosed, high-risk, locally advanced cervical cancer (ENGOT-cx11/GOG-3047/KEYNOTE-A18): a randomised, double-blind, phase 3 clinical trial. Lancet. (2024) 403:1341–50. doi: 10.1016/s0140-6736(24)00317-9 38521086

[17] Ghisoni E Morotti M Sarivalasis A Grimm AJ Kandalaft L Laniti DD . Immunotherapy for ovarian cancer: towards a tailored immunophenotype-based approach. Nat Rev Clin Oncol. (2024) 21:801–17. doi: 10.1038/s41571-024-00937-4 39232212

[18] Wan C Keany MP Dong H Al-Alem LF Pandya UM Lazo S . Enhanced efficacy of simultaneous PD-1 and PD-L1 immune checkpoint blockade in high-grade serous ovarian cancer. Cancer Res. (2021) 81:158–73. doi: 10.1158/0008-5472.can-20-1674 33158814 PMC7878408

[19] Dvorkin S Cambier S Volkman HE Stetson DB . New frontiers in the cGAS-STING intracellular DNA-sensing pathway. Immunity. (2024) 57:718–30. doi: 10.1016/j.immuni.2024.02.019 38599167 PMC11013568

[20] Li Q Song Q Ma L Kang K Zhu X Li Y . The cGAS-STING pathway in cancer: friend or foe. Cell Death Dis. (2026) 17:374. doi: 10.1038/s41419-026-08607-2 41881950 PMC13040067

[21] Yu L Liu P . Cytosolic DNA sensing by cGAS: regulation, function, and human diseases. Signal Transduction Targeted Ther. (2021) 6:170. doi: 10.1038/s41392-021-00554-y 33927185 PMC8085147

[22] Han J Hu S Hu Y Xu Y Hou Y Yang Y . Discovery of podofilox as a potent cGAMP-STING signaling enhancer with antitumor activity. Cancer Immunol Res. (2023) 11:583–99. doi: 10.1158/2326-6066.cir-22-0483 36921097

[23] Sun L Wu J Du F Chen X Chen ZJ . Cyclic GMP-AMP synthase is a cytosolic DNA sensor that activates the type I interferon pathway. Science. (2013) 339:786–91. doi: 10.1126/science.1232458 23258413 PMC3863629

[24] Cui S Yu Q Chu L Cui Y Ding M Wang Q . Nuclear cGAS functions non-canonically to enhance antiviral immunity via recruiting methyltransferase Prmt5. Cell Rep. (2020) 33:108490. doi: 10.1016/j.celrep.2020.108490 33296647

[25] Zhao T Zhao T Dong D Dong D . Genome doubling as a dynamic driver of ovarian cancer evolution: insights from single-cell sequencing. J Ovarian Res. (2025) 18:274. doi: 10.1186/s13048-025-01860-7 41257949 PMC12628590

[26] Woo S-R Fuertes MB Corrales L Spranger S Furdyna MJ Leung MY . STING-dependent cytosolic DNA sensing mediates innate immune recognition of immunogenic tumors. Immunity. (2014) 41:830–42. doi: 10.1016/j.immuni.2014.10.017 25517615 PMC4384884

[27] Liang S Ma H Liu Y Hai L Tian Y Sun Y . Nano-immunomodulator amplifies STING activation in tumor-associated macrophages for cancer immunotherapy. J Control Release. (2025) 383:113846. doi: 10.1016/j.jconrel.2025.113846 40379214

[28] Wang L Zhu S Ding Z Hong J Zheng L Sun J . Cell-type specific activation of the cGAS-STING pathway in tumor immunotherapy: mechanisms and therapeutic implications. J Transl Med. (2026) 24:43. doi: 10.1186/s12967-025-07645-2 41501823 PMC12790129

[29] Tijhuis AE Johnson SC McClelland SE . The emerging links between chromosomal instability (CIN), metastasis, inflammation and tumour immunity. Mol Cytogenet. (2019) 12:17. doi: 10.1186/s13039-019-0429-1 31114634 PMC6518824

[30] Wu J Sun L Chen X Du F Shi H Chen C . Cyclic GMP-AMP is an endogenous second messenger in innate immune signaling by cytosolic DNA. Science. (2013) 339:826–30. doi: 10.1126/science.1229963 23258412 PMC3855410

[31] Michalski S de Oliveira Mann CC Stafford CA Witte G Bartho J Lammens K . Structural basis for sequestration and autoinhibition of cGAS by chromatin. Nature. (2020) 587:678–82. doi: 10.1038/s41586-020-2748-0 32911480

[32] Boyer JA Spangler CJ Strauss JD Cesmat AP Liu P McGinty RK . Structural basis of nucleosome-dependent cGAS inhibition. Science. (2020) 370:450–4. doi: 10.1126/science.abd0609 32913000 PMC8189757

[33] Zhao B Xu P Rowlett CM Jing T Shinde O Lei Y . The molecular basis of tight nuclear tethering and inactivation of cGAS. Nature. (2020) 587:673–7. doi: 10.1107/s2053273323086783 32911481 PMC7704945

[34] Sun Y Patterson-Fortin J Han S Li Z Nowicka Z Hirohashi Y . 53BP1 loss elicits cGAS-STING-dependent antitumor immunity in ovarian and pancreatic cancer. Nat Commun. (2024) 15:6676. doi: 10.1038/s41467-024-50999-2 39107288 PMC11303708

[35] Dou Z Kreiling JA Heynen-Genel S Jurk D Neretti N Adams PD . Cytosolic DNA crosstalk in senescence: a new axis of inflammatory signaling? EMBO J. (2025) 44:5239–43. doi: 10.1038/s44318-025-00531-z 40883506 PMC12489115

[36] Kandhaya-Pillai R Kim HY Belhadj J Sidorova JM Tchkonia T Kirkland JL . TRF2 enriched cytoplasmic chromatin drives cGAS-STAT1-mediated inflammation in senescence. iScience. (2026) 29:116770. doi: 10.1016/j.isci.2026.116770 42519061 PMC13382140

[37] Ying X Chen Q Yang Y Wu Z Zeng W Miao C . Nanomedicines harnessing cGAS-STING pathway: sparking immune revitalization to transform ‘cold’ tumors into ‘hot’ tumors. Mol Cancer. (2024) 23:277. doi: 10.1186/s12943-024-02186-6 39710707 PMC11664935

[38] Sun R Jiang P Wang Z Yan B Zhu S Huang Z . DNA2 protein destruction dictates DNA hyperexcision, cGAS-STING activation, and innate immune response in CDK12-deregulated cancers. Proc Natl Acad Sci USA. (2025) 122:e2413732122. doi: 10.1073/pnas.2413732122 40623188 PMC12280927

[39] Zhang Z Zhou H Ouyang X Dong Y Sarapultsev A Luo S . Multifaceted functions of STING in human health and disease: from molecular mechanism to targeted strategy. Signal Transduction Targeted Ther. (2022) 7:394. doi: 10.1038/s41392-022-01252-z 36550103 PMC9780328

[40] Yum S Li M Fang Y Chen ZJ . TBK1 recruitment to STING activates both IRF3 and NF-κB that mediate immune defense against tumors and viral infections. Proc Natl Acad Sci. (2021) 118:e2100225118. doi: 10.1073/pnas.2100225118 33785602 PMC8040795

[41] Zhao B Du F Xu P Shu C Sankaran B Bell SL . A conserved PLPLRT/SD motif of STING mediates the recruitment and activation of TBK1. Nature. (2019) 569:718–22. doi: 10.1038/s41586-019-1228-x 31118511 PMC6596994

[42] Hopfner K-P Hornung V . Molecular mechanisms and cellular functions of cGAS–STING signalling. Nat Rev Mol Cell Biol. (2020) 21:501–21. doi: 10.1038/s41580-020-0244-x 32424334

[43] Lanng KRB Lauridsen EL Jakobsen MR . The balance of STING signaling orchestrates immunity in cancer. Nat Immunol. (2024) 25:1144–57. doi: 10.1038/s41590-024-01872-3 38918609

[44] Fahey CG Cordova AF Gedeon PC Barbie DA . Targeting STING to generate therapeutic anti-tumor immunity. Cancer Cell. (2026) 44:260–80. doi: 10.1016/j.ccell.2025.12.002 41448179 PMC12779296

[45] Zhang L Wei X Wang Z Liu P Hou Y Xu Y . NF-κB activation enhances STING signaling by altering microtubule-mediated STING trafficking. Cell Rep. (2023) 42:112185. doi: 10.1016/j.celrep.2025.116853 36857187

[46] ZhangB PedersenA ReinertLS LiY NaritaR IdornM . STING signals to NF-κB from late endolysosomal compartments using IRF3 as an adaptor. Nat Immunol. (2025) 26:1916–30. doi: 10.1038/s41590-025-02283-840973797

[47] Salas-Benito D Vercher E Conde E Glez-Vaz J Tamayo I Hervas-Stubbs S . Inflammation and immunity in ovarian cancer. Eur J Cancer Suppl. (2020) 15:56–66. doi: 10.1016/j.ejcsup.2019.12.002 33240443 PMC7569134

[48] Jordan KR Sikora MJ Slansky JE Minic A Richer JK Moroney MR . The capacity of the ovarian cancer tumor microenvironment to integrate inflammation signaling conveys a shorter disease-free interval. Clin Cancer Res. (2020) 26:6362–73. doi: 10.1158/1078-0432.ccr-20-1762 32928797 PMC7923250

[49] Guo Y Jiang F Kong L Li B Yang Y Zhang L . Cutting edge: USP27X deubiquitinates and stabilizes the DNA sensor cGAS to regulate cytosolic DNA-mediated signaling. J Immunol. (2019) 203:2049–54. doi: 10.4049/jimmunol.1900514 31534008

[50] Qin Y Wang M Meng X Wang M Jiang H Gao Y . ISGylation by HERCs facilitates STING activation. Cell Rep. (2024) 43:114135. doi: 10.1016/j.celrep.2024.114135 38652662

[51] Kim DS Park Y Tsokos GC Cho M-L Kwok S-K . The ubiquitin E3 ligase TRIM21 suppresses type I interferon signaling via STING degradation and ameliorates systemic autoimmunity. Exp Mol Med. (2025) 57:1555–66. doi: 10.1038/s12276-025-01490-5 40610751 PMC12322227

[52] Yao S Chen S Wang A Liang Z Liu X Gao Y . BAG2 inhibits cervical cancer progression by modulating type I interferon signaling through stabilizing STING. Adv Sci (Weinh). (2025) 12:e70005. doi: 10.1002/advs.202414637 40364789 PMC12362813

[53] Zhang L Wei N Cui Y Hong Z Liu X Wang Q . The deubiquitinase CYLD is a specific checkpoint of the STING antiviral signaling pathway. PloS Pathog. (2018) 14:e1007435. doi: 10.1371/journal.ppat.1007435 30388174 PMC6235404

[54] Wang J Wang Z Wan S Ye S Li Q Liu Y . Lumpy skin disease virus ORF142 suppresses the cGAS/STING-mediated IFN-I pathway through NBR1-mediated STING autophagic degradation. Microb Pathogen. (2025) 208:107959. doi: 10.1016/j.micpath.2025.107959 40774565

[55] Zhang R Yu C Zeh HJ Kroemer G Klionsky DJ Tang D . TAX1BP1-dependent autophagic degradation of STING1 impairs anti-tumor immunity. Autophagy. (2025) 21:1802–23. doi: 10.1080/15548627.2025.2471736 40000606 PMC12283022

[56] Zhao Y-J Bai H-Z Wang Y-N Liu Y-K Zhao L-B Li Z . GABAergic disinhibition through the STING-dependent autophagic pathway in the dorsal horn underlies diabetic neuropathic pain pathogenesis. Neurosci Lett. (2025) 868:138391. doi: 10.1016/j.neulet.2025.138391 41027494

[57] de Queiroz N Xia T Konno H Barber GN . Ovarian cancer cells commonly exhibit defective STING signaling which affects sensitivity to viral oncolysis. Mol Cancer Res. (2019) 17:974–86. doi: 10.1158/1541-7786.mcr-18-0504 30587523 PMC6445711

[58] Liu J Liu C Ma Y Pan X Chu R Yao S . STING inhibitors sensitize platinum chemotherapy in ovarian cancer by inhibiting the CGAS-STING pathway in cancer-associated fibroblasts (CAFs). Cancer Lett. (2024) 588:216700. doi: 10.1016/j.canlet.2024.216700 38373690

[59] Zhang B Laursen MF Hu L Hazrati H Narita R Jensen LS . Cholesterol-binding motifs in STING that control endoplasmic reticulum retention mediate anti-tumoral activity of cholesterol-lowering compounds. Nat Commun. (2024) 15:2760. doi: 10.1038/s41467-024-47046-5 38553448 PMC10980718

[60] Kanoda R Nakajima S Okayama H Kaneta A Chida S Matsumoto T . Downregulation of cGAS/STING expression in tumor cells by cancer-associated fibroblasts in colorectal cancer. Sci Rep. (2025) 15:19234. doi: 10.1038/s41598-025-03924-6 40451897 PMC12127464

[61] Liang J-L Jin X-K Zhang S-M Huang Q-X Ji P Deng X-C . Specific activation of cGAS-STING pathway by nanotherapeutics-mediated ferroptosis evoked endogenous signaling for boosting systemic tumor immunotherapy. Sci Bull. (2023) 68:622–36. doi: 10.1016/j.scib.2023.02.027 36914548

[62] Gao Y Zheng X Chang B Lin Y Huang X Wang W . Intercellular transfer of activated STING triggered by RAB22A-mediated non-canonical autophagy promotes antitumor immunity. Cell Res. (2022) 32:1086–104. doi: 10.1038/s41422-022-00731-w 36280710 PMC9715632

[63] Hosea R Hillary S Naqvi S Wu S Kasim V . The two sides of chromosomal instability: drivers and brakes in cancer. Signal Transduction Targeted Ther. (2024) 9:75. doi: 10.1038/s41392-024-01767-7 38553459 PMC10980778

[64] Harris EJ Karaoglu DA Sukhanova M Abaza Y Karantanos T Eisfeld A-K . Clinical and molecular characterization of TP53-mutant acute lymphoblastic leukemia in adults. Blood Cancer J. (2025) 15:138. doi: 10.1038/s41408-025-01350-5 40813576 PMC12354737

[65] Light N Layeghifard M Attery A Subasri V Zatzman M Anderson ND . Germline TP53 mutations undergo copy number gain years prior to tumor diagnosis. Nat Commun. (2023) 14:77. doi: 10.1038/s41467-022-35727-y 36604421 PMC9816166

[66] Agustinus AS Al-Rawi D Dameracharla B Raviram R Jones BSCL Stransky S . Epigenetic dysregulation from chromosomal transit in micronuclei. Nature. (2023) 619:176–83. doi: 10.1038/s41586-023-06084-7 37286593 PMC10322720

[67] Hong C Schubert M Tijhuis AE Requesens M Roorda M van den Brink A . cGAS-STING drives the IL-6-dependent survival of chromosomally instable cancers. Nature. (2022) 607:366–73. doi: 10.1038/s41586-022-04847-2 35705809

[68] Aguilera A Gómez-González B . Genome instability: a mechanistic view of its causes and consequences. Nat Rev Genet. (2008) 9:204–17. doi: 10.1038/nrg2268 18227811

[69] Bell D Berchuck A Birrer M Chien J Cramer DW Dao F . Integrated genomic analyses of ovarian carcinoma. Nature. (2011) 474:609–15. doi: 10.1038/nature10166 PMC3163504 21720365

[70] Ewing A Meynert A Churchman M Grimes GR Hollis RL Herrington CS . Structural variants at the BRCA1/2 loci are a common source of homologous repair deficiency in high-grade serous ovarian carcinoma. Clin Cancer Res. (2021) 27:3201–14. doi: 10.1158/1078-0432.ccr-20-4068 33741650 PMC7610896

[71] Hu Q Espejo Valle-Inclán J Dahiya R Guyer A Mazzagatti A Maurais EG . Non-homologous end joining shapes the genomic rearrangement landscape of chromothripsis from mitotic errors. Nat Commun. (2024) 15:5611. doi: 10.1101/2023.08.10.552800 38965240 PMC11224358

[72] Incorvaia L Bazan Russo TD Gristina V Perez A Brando C Mujacic C . The intersection of homologous recombination (HR) and mismatch repair (MMR) pathways in DNA repair-defective tumors. NPJ Precis Oncol. (2024) 8:190. doi: 10.1038/s41698-024-00672-0 39237751 PMC11377838

[73] McKenney CD Regot S . Mechanisms of whole-genome doubling in cancer evolution. Trends Cancer. (2026) 12:175–85. doi: 10.1016/j.trecan.2025.10.001 41206259 PMC12784356

[74] Dewhurst SM McGranahan N Burrell RA Rowan AJ Grönroos E Endesfelder D . Tolerance of whole-genome doubling propagates chromosomal instability and accelerates cancer genome evolution. Cancer Discov. (2014) 4:175–85. doi: 10.1158/2159-8290.cd-13-0285 24436049 PMC4293454

[75] Li J Hubisz MJ Earlie EM Duran MA Hong C Varela AA . Non-cell-autonomous cancer progression from chromosomal instability. Nature. (2023) 620:1080–8. doi: 10.1038/s41586-023-06464-z 37612508 PMC10468402

[76] Quinton RJ DiDomizio A Vittoria MA Kotýnková K Ticas CJ Patel S . Whole-genome doubling confers unique genetic vulnerabilities on tumour cells. Nature. (2021) 590:492–7. doi: 10.1038/s41586-020-03133-3 33505027 PMC7889737

[77] Mackenzie KJ Carroll P Martin CA Murina O Fluteau A Simpson DJ . cGAS surveillance of micronuclei links genome instability to innate immunity. Nature. (2017) 548:461–5. doi: 10.1038/nature23449 28738408 PMC5870830

[78] Harding SM Benci JL Irianto J Discher DE Minn AJ Greenberg RA . Mitotic progression following DNA damage enables pattern recognition within micronuclei. Nature. (2017) 548:466–70. doi: 10.1038/nature23470 28759889 PMC5857357

[79] Bakhoum SF Ngo B Laughney AM Cavallo JA Murphy CJ Ly P . Chromosomal instability drives metastasis through a cytosolic DNA response. Nature. (2018) 553:467–72. doi: 10.1016/j.ijrobp.2018.06.295 29342134 PMC5785464

[80] Xu Z Xie H Song L Huang Y Huang J . BRCA1 and BRCA2 in DNA damage and replication stress response: Insights into their functions, mechanisms, and implications for cancer treatment. DNA Repair. (2025) 150:103847. doi: 10.1016/j.dnarep.2025.103847 40373656

[81] Quaas M Kohler R Nöltner L Schmidbauer LF Uxa S Müller GA . BRCA1 and BRCA2 gene expression: p53- and cell cycle-dependent repression requires RB and DREAM. Cell Death Differ. (2026) 33:51–63. doi: 10.1038/s41418-025-01566-9 40841483 PMC12811384

[82] Madaan V Kollara A Spaner D Brown TJ . BRCA1 deficiency induces NFκB-dependent type-I interferon signaling in fallopian tube epithelial cells. Neoplasia. (2026) 78:101320. doi: 10.1016/j.neo.2026.101320 42190606 PMC13223825

[83] Datta A Jackson J Morozov YI Qiu J Vindigni A Greenberg RA . The BRCA1-A complex restricts replication fork reversal-dependent DNA repair in ATM deficient cells. Nat Commun. (2026) 17. doi: 10.1038/s41467-026-75271-7 42401588 PMC13470386

[84] Bruand M Barras D Mina M Ghisoni E Morotti M Lanitis E . Cell-autonomous inflammation of BRCA1-deficient ovarian cancers drives both tumor-intrinsic immunoreactivity and immune resistance via STING. Cell Rep. (2021) 36:109412. doi: 10.1016/j.celrep.2021.109412 34289354 PMC8371260

[85] Kraya AA Maxwell KN Wubbenhorst B Wenz BM Pluta J Rech AJ . Genomic signatures predict the immunogenicity of BRCA-deficient breast cancer. Clin Cancer Res. (2019) 25:4363–74. doi: 10.1158/1078-0432.ccr-18-0468 30914433 PMC6635013

[86] Samstein RM Krishna C Ma X Pei X Lee K-W Makarov V . Mutations in BRCA1 and BRCA2 differentially affect the tumor microenvironment and response to checkpoint blockade immunotherapy. Nat Cancer. (2020) 1:1188–203. doi: 10.1038/s43018-020-00139-8 33834176 PMC8023400

[87] Feng C Wu D Li H Ma D Chen G Sun C . 5P type I interferon niche drives immune suppression by CD8+ T cell dysfunction in ovarian cancer. Ann Oncol. (2025) 36:S209. doi: 10.1016/j.annonc.2025.08.44242574925

[88] van Vugt MATM Parkes EE . When breaks get hot: inflammatory signaling in BRCA1/2-mutant cancers. Trends Cancer. (2022) 8:174–89. doi: 10.1016/j.trecan.2021.12.003 35000881

[89] Sokol ES Pavlick D Frampton GM Ross JS Miller VA Ali SM . Pan-cancer analysis of CDK12 loss-of-function alterations and their association with the focal tandem-duplicator phenotype. Oncologist. (2019) 24:1526–33. doi: 10.1634/theoncologist.2019-0214 31292271 PMC6975947

[90] Curti L Rohban S Bianchi N Croci O Andronache A Barozzi S . CDK12 controls transcription at damaged genes and prevents MYC-induced transcription-replication conflicts. Nat Commun. (2024) 15:7100. doi: 10.1038/s41467-024-51229-5 39155303 PMC11330984

[91] Tien JC Zhai Y Wu R Zhang Y Chang Y Cheng Y . Defining CDK12 as a tumor suppressor and therapeutic target in mouse models of tubo-ovarian high-grade serous carcinoma. Proc Natl Acad Sci USA. (2025) 122:e2426909122. doi: 10.1073/pnas.2426909122 40504161 PMC12184368

[92] Lenaerts L Reynhout S Verbinnen I Laumonnier F Toutain A Bonnet-Brilhault F . The broad phenotypic spectrum of PPP2R1A-related neurodevelopmental disorders correlates with the degree of biochemical dysfunction. Genet Med. (2021) 23:352–62. doi: 10.1038/s41436-020-00981-2 33106617 PMC7862067

[93] Mondal I Das O Sun R Gao J Yu B Diaz A . PP2Ac deficiency enhances tumor immunogenicity by activating STING–type I interferon signaling in glioblastoma. Cancer Res. (2023) 83:2527–42. doi: 10.1158/0008-5472.can-22-3382 37219874 PMC10525036

[94] Brodeur MN Dopeso H Zhu Y Longhini ALF Gazzo A Sun S . Interferon response and epigenetic modulation by SMARCA4 mutations drive ovarian tumor immunogenicity. Sci Adv. (2024) 10:eadk4851. doi: 10.1101/2023.08.08.552544 39630912 PMC11616711

[95] Yang H-J Kim E-J Kim S Song S-H Kim T-Y . SMARCA4 activation engages FOSL1 to drive enhancer reprogramming and tumorigenic phenotypes in SMARCA4-deficient LUAD cells. Cell Death Discov. (2026) 12:262. doi: 10.1038/s41420-026-03100-3 42010258 PMC13223256

[96] Reen V D’Ambrosio M Søgaard PP Tyson K Leeke BJ Clément I . SMARCA4 regulates the NK-mediated killing of senescent cells. Sci Adv. (2025) 11:eadn2811. doi: 10.1126/sciadv.adn2811 39813356 PMC11734740

[97] Lopez-Pelaez M Young L Vazquez-Chantada M Nelson N Durant S Wilkinson RW . Targeting DNA damage response components induces enhanced STING-dependent type-I IFN response in ATM deficient cancer cells and drives dendritic cell activation. Oncoimmunology. (2022) 11:2117321. doi: 10.1080/2162402x.2022.2117321 36117525 PMC9481087

[98] Pantelidou C Sonzogni O De Oliveria Taveira M Mehta AK Kothari A Wang D . PARP inhibitor efficacy depends on CD8(+) T-cell recruitment via intratumoral STING pathway activation in BRCA-deficient models of triple-negative breast cancer. Cancer Discov. (2019) 9:722–37. doi: 10.1158/2159-8290.cd-18-1218 31015319 PMC6548644

[99] Ding L Kim HJ Wang Q Kearns M Jiang T Ohlson CE . PARP inhibition elicits STING-dependent antitumor immunity in Brca1-deficient ovarian cancer. Cell Rep. (2018) 25:2972–2980.e5. doi: 10.1016/j.celrep.2018.11.054 30540933 PMC6366450

[100] Mao C Fan W Liu J Yang F Li W Li L . Targeting HDAC and PARP enhances STING-dependent antitumor immunity in STING-deficient tumor. Adv Sci (Weinh). (2025) 12:e07904. doi: 10.1002/advs.202507904 40789065 PMC12591105

[101] Shen J Zhao W Ju Z Wang L Peng Y Labrie M . PARPi triggers the STING-dependent immune response and enhances the therapeutic efficacy of immune checkpoint blockade independent of BRCAness. Cancer Res. (2019) 79:311–9. doi: 10.1158/0008-5472.can-18-1003 30482774 PMC6588002

[102] Speak A Hijaz M Singh H Udumula M Miller M Munkarah A . Inhibiting mitochondria potentiates PARPi-triggered, STING-dependent immune response in pre-clinical models of epithelial ovarian cancer. Gynecologic Oncol. (2024) 190:S233–4. doi: 10.1016/j.ygyno.2024.07.33842574925

[103] Humer D Klepsch V Baier G . Potentiation of cancer immunogenicity by targeting PARP. J Immunother Cancer. (2025) 13:e011056. doi: 10.1136/jitc-2024-011056 40555563 PMC12198780

[104] Ding L Wang Q Martincuks A Kearns MJ Jiang T Lin Z . STING agonism overcomes STAT3-mediated immunosuppression and adaptive resistance to PARP inhibition in ovarian cancer. J Immunother Cancer. (2023) 11:e005627. doi: 10.1136/jitc-2022-005627 36609487 PMC9827255

[105] Zhu Q Dai Q Zhao L Zheng C Li Q Yuan Z . Novel dual inhibitors of PARP and HDAC induce intratumoral STING-mediated antitumor immunity in triple-negative breast cancer. Cell Death Dis. (2024) 15:10. doi: 10.1038/s41419-023-06303-z 38182579 PMC10770036

[106] Gronauer R Madersbacher L Monfort-Lanzas P Floriani G Sprung S Zeimet AG . Integrated immunogenomic analyses of high-grade serous ovarian cancer reveal vulnerability to combination immunotherapy. Front Immunol. (2024) 15:1489235. doi: 10.3389/fimmu.2024.1489235 39669575 PMC11634877

[107] Konstantinopoulos PA Waggoner S Vidal GA Mita M Moroney JW Holloway R . Single-arm phases 1 and 2 trial of niraparib in combination with pembrolizumab in patients with recurrent platinum-resistant ovarian carcinoma. JAMA Oncol. (2019) 5:1141–9. doi: 10.1001/jamaoncol.2019.1048 31194228 PMC6567832

[108] Monk BJ Colombo N Oza AM Fujiwara K Birrer MJ Randall L . Chemotherapy with or without avelumab followed by avelumab maintenance versus chemotherapy alone in patients with previously untreated epithelial ovarian cancer (JAVELIN Ovarian 100): an open-label, randomised, phase 3 trial. Lancet Oncol. (2021) 22:1275–89. doi: 10.1016/s1470-2045(21)00342-9 34363762

[109] Shen J Xu F Liu T Ye Y Xu S . NAD+ metabolism-mediated SURF4-STING axis enhances T-cell anti-tumor effects in the ovarian cancer microenvironment. Cell Death Dis. (2025) 16:640. doi: 10.1038/s41419-025-07939-9 40846839 PMC12373823

[110] Liu X Yao Q Wang Z Wang P Zhang Z Shen Z . POSTN(+) CAFs facilitate gastric cancer peritoneal metastasis by promoting ICAM-1-dependent tumor cell adhesion and CD8(+) T-cell exhaustion. Front Immunol. (2026) 17:1796080. doi: 10.3389/fimmu.2026.1796080 42358949 PMC13291120

[111] Bai Y Chen R Lu X . A CAF-associated stromal remodeling signature links immune exclusion to exhaustion-prone CD8(+) T-cell dysfunction in high-grade serous ovarian cancer. Int J Mol Sci. (2026) 27:6092. doi: 10.3390/ijms27136092 42450357 PMC13361325

[112] Huang Z Zhu J Zhou YL Shi J . The cGAS-STING pathway: a dual regulator of immune response in cancer and therapeutic implications. J Transl Med. (2025) 23:766. doi: 10.1186/s12967-025-06843-2 40640925 PMC12247247

[113] Hou L Zhang M Tong W Zhang S . PARylation-centric crosstalk: orchestrating immune evasion and multidrug resistance in ovarian cancer. Front Immunol Volume 17 - 2026. (2026) 17. doi: 10.3389/fimmu.2026.1772562 42148103 PMC13176232

[114] Zou Y Zhang H Chen P Tang J Yang S Nicot C . Clinical approaches to overcome PARP inhibitor resistance. Mol Cancer. (2025) 24:156. doi: 10.1186/s12943-025-02355-1 40442774 PMC12123805

[115] Concannon K Morris BB Gay CM Byers LA . Combining targeted DNA repair inhibition and immune-oncology approaches for enhanced tumor control. Mol Cell. (2023) 83:660–80. doi: 10.1016/j.molcel.2022.12.031 36669489 PMC9992136

[116] Traboulsi W Gaur P Kundu S Ramlaoui Z Priestley-Milianta C Iyengar A . PARP inhibition generates enhanced CD8+ central memory T cells by transcriptional and metabolic reprogramming. Nat Immunol. (2026) 27:189–99. doi: 10.1038/s41590-025-02383-5 41526551

[117] Basavaraja R Zhang H Holczbauer Á Lu Z Radaelli E Assenmacher C-A . PARP11 inhibition inactivates tumor-infiltrating regulatory T cells and improves the efficacy of immunotherapies. Cell Rep Med. (2024) 5:101649. doi: 10.1016/j.xcrm.2024.101649 39019005 PMC11293321

[118] Stange C Dreyer TF Riedel M Elsen F Seitz S Hamann D . PARP inhibitor-induced anti-tumour chemokine response is suppressed by dipeptidyl peptidase 4 (DPP4) in ovarian cancer. Br J Cancer. (2025) 133:582–93. doi: 10.1038/s41416-025-03076-4 40579444 PMC12356936

[119] Ran X Zhang X Teng M Alawi WB Nahum S He H . The effect of PARP inhibitor radiosensitization on the mRNA translational regulation of T cell chemokines. Int J Radiat Oncol Biol Phys. (2023) 117:S71–2. doi: 10.1016/j.ijrobp.2023.06.38042574925

[120] Bustos MA Yokoe T Shoji Y Kobayashi Y Mizuno S Murakami T . MiR-181a targets STING to drive PARP inhibitor resistance in BRCA- mutated triple-negative breast cancer and ovarian cancer. Cell Biosci. (2023) 13:200. doi: 10.1186/s13578-023-01151-y 37932806 PMC10626784

[121] Guo E Xiao R Wu Y Lu F Liu C Yang B . WEE1 inhibition induces anti-tumor immunity by activating ERV and the dsRNA pathway. J Exp Med. (2022) 219:e20210789. doi: 10.1084/jem.20210789 34825915 PMC8628262

[122] Sun L Moore E Berman R Clavijo PE Saleh A Chen Z . WEE1 kinase inhibition reverses G2/M cell cycle checkpoint activation to sensitize cancer cells to immunotherapy. Oncoimmunology. (2018) 7:e1488359. doi: 10.1080/2162402x.2018.1488359 30288354 PMC6169577

[123] Jiao X Liu J Wu Y Zhong Q Zhu L Wang L . Sequential treatment with PARPi and WEE1i enhances antitumor immune responses in preclinical models of ovarian cancer. Sci Transl Med. (2025) 17:eadu6989. doi: 10.1126/scitranslmed.adu6989 40802736

[124] Giudice E Huang T-T Nair JR Zurcher G McCoy A Nousome D . The CHK1 inhibitor prexasertib in BRCA wild-type platinum-resistant recurrent high-grade serous ovarian carcinoma: a phase 2 trial. Nat Commun. (2024) 15:2805. doi: 10.1038/s41467-024-47215-6 38555285 PMC10981752

[125] Chen J-Y Ke T-W Chiang S-F Hong W-Z Chang H-Y Liang J-A . CHK1 inhibition increases the therapeutic response to radiotherapy via antitumor immunity in ARID1A-deficient colorectal cancer. Cell Death Dis. (2025) 16:584. doi: 10.1038/s41419-025-07912-6 40750787 PMC12317038

[126] Zhang J Xiang F Hu Y Wang J Wang T Zhang Z . CHK1 inhibition by prexasertib sensitizes cisplatin-resistant malignant tumor cells via checkpoint abrogation and STAT1-driven PD-L1 upregulation. Int Immunopharmacol. (2026) 171:116126. doi: 10.1016/j.intimp.2025.116126 41477994

[127] Chen M Huang L Zhu M Cai J Ying F Liu L . Combined CHK1 and PD-L1 blockade as a novel therapeutic strategy against stemness and immunosuppression in ovarian cancer. Cancer Immunol Immunother. (2025) 74:365. doi: 10.1007/s00262-025-04215-9 41196428 PMC12592612

[128] Liu S Deng P Yu Z Hong JH Gao J Huang Y . CDC7 inhibition potentiates antitumor efficacy of PARP inhibitor in advanced ovarian cancer. Adv Sci. (2024) 11:2403782. doi: 10.1002/advs.202403782 39412086 PMC11615783

[129] Nespolo A Stefenatti L Pellarin I Gambelli A Rampioni Vinciguerra GL Karimbayli J . USP1 deubiquitinates PARP1 to regulate its trapping and PARylation activity. Sci Adv. (2024) 10:eadp6567. doi: 10.1126/sciadv.adp6567 39536107 PMC11559621

[130] Han D Wang L Jiang S Su P Chen B Zhao W . The USP1-WDR48 deubiquitinase complex functions as a molecular switch regulating tumor-associated macrophage activation and anti-tumor response. Cell Death Differ. (2026) 33:92–110. doi: 10.1038/s41418-025-01548-x 40707784 PMC12811369

[131] Zhou W Zhao Y Qin W Wu W Liao C Zhang Y . Targeting USP1 potentiates radiation-induced type I IFN-dependent antitumor immunity by enhancing oligo-ubiquitinated SAR1A-mediated STING trafficking and activation. Adv Sci (Weinh). (2025) 12:e2412687. doi: 10.1002/advs.202412687 39976106 PMC12005740

[132] Zhang H Sun Y Zhang L Shen M Tong X Zhao D . Polymer-PARPi conjugates delivering USP1i for maximizing synthetic lethality to stimulate STING pathway in high-grade serous ovarian cancer. Adv Mater. (2026) 38:e12962. doi: 10.1002/adma.202512962 40970784

[133] Shim A Chen Y Maciejowski J . Activation and regulation of cGAS-STING signaling in cancer cells. Mol Cell. (2025) 85:3807–22. doi: 10.1016/j.molcel.2025.08.030 41106370 PMC12614337

[134] Wang Y Yue B Ni H Chen J Shi R Wang Z . POSTN^+^ cancer-associated fibroblast–CCL3^+^ macrophage crosstalk defines the immune-excluded tumor microenvironment in clear cell renal cell carcinoma. Transl Oncol. (2026) 65:102682. doi: 10.1016/j.tranon.2026.102682 41579565 PMC12860634

[135] Zhang C Qian C Lv W Zhang Q Wang Y . POSTN(+) fibroblast-secreted small extracellular vesicles drive macrophage M2 polarization through BMP4/BMPR2/Smad signaling. Oncol Rep. (2026) 55:62. doi: 10.3892/or.2026.9067 41645751 PMC12891935

[136] Jiang H Li Z Li X Yu L Sun J Shen Y . The cGAS–STING pathway in tumor immunity: dual roles, regulatory mechanisms, and precision therapeutic strategies. Front Pharmacol Volume 17 - 2026. (2026) 17. doi: 10.3389/fphar.2026.1844637 42488575 PMC13388410

[137] Jang SC Economides KD Moniz RJ Sia CL Lewis N McCoy C . ExoSTING, an extracellular vesicle loaded with STING agonists, promotes tumor immune surveillance. Commun Biol. (2021) 4:497. doi: 10.1038/s42003-021-02004-5 33888863 PMC8062530

[138] Li G Zhao X Zheng Z Zhang H Wu Y Shen Y . cGAS-STING pathway mediates activation of dendritic cell sensing of immunogenic tumors. Cell Mol Life Sci. (2024) 81:149. doi: 10.1007/s00018-024-05191-6 38512518 PMC10957617

[139] Ng I-I Zhang Z Xiao K Ye M Tian T Zhu Y . Targeting WEE1 in tumor-associated dendritic cells potentiates antitumor immunity via the cGAS/STING pathway. Cell Rep. (2025) 44:115733. doi: 10.1016/j.celrep.2025.115733 40397571

[140] Wang X Liu Y Xue C Hu Y Zhao Y Cai K . A protein-based cGAS-STING nanoagonist enhances T cell-mediated anti-tumor immune responses. Nat Commun. (2022) 13:5685. doi: 10.1038/s41467-022-33301-0 36167857 PMC9515186

[141] Wang X Wang X Li H Liu S Lu Y Chen H . tRNA m1A modification orchestrates STING translation in macrophages to enhance antitumor immunity and CAR-macrophage immunotherapy. Cell Mol Immunol. (2026) 23:261–72. doi: 10.1038/s41423-025-01383-7 41535541 PMC12949049

[142] Wang Q Bergholz JS Ding L Lin Z Kabraji SK Hughes ME . STING agonism reprograms tumor-associated macrophages and overcomes resistance to PARP inhibition in BRCA1-deficient models of breast cancer. Nat Commun. (2022) 13:3022. doi: 10.1038/s41467-022-30568-1 35641483 PMC9156717

[143] Li M Ma Y Dai T Wang Y Yue Y . Advance in therapies targeting tumor-associated macrophages in ovarian cancer. Front Immunol Volume 16 - 2025. (2025) 16. doi: 10.3389/fimmu.2025.1677839 40963633 PMC12439345

[144] Li K Shi H Zhang B Ou X Ma Q Chen Y . Myeloid-derived suppressor cells as immunosuppressive regulators and therapeutic targets in cancer. Signal Transduction Targeted Ther. (2021) 6:362. doi: 10.1038/s41392-021-00670-9 34620838 PMC8497485

[145] Wu L Deng Z Peng Y Han L Liu J Wang L . Ascites-derived IL-6 and IL-10 synergistically expand CD14(+)HLA-DR(-/low) myeloid-derived suppressor cells in ovarian cancer patients. Oncotarget. (2017) 8:76843–56. doi: 10.18632/oncotarget.20164 29100353 PMC5652747

[146] Rivera-López YA Torres-Rosado AP Casiano-Martínez JA Meléndez-Rodríguez LL Imad-Hamad R Hernández-Carrasquillo SM . Chronic stress drives ovarian cancer progression via myeloid-derived suppressor cells infiltration and Notch signaling pathway activation. Front Immunol. (2025) 16:1593299. doi: 10.3389/fimmu.2025.1593299 41488655 PMC12757280

[147] Manda SS Espersen MM Mapagu C Bouantoun N Boros J Chiew Y-E . Overcoming intra-tumoral heterogeneity for biomarker discovery in the high-grade serous ovarian cancer proteome. NPJ Precis Oncol. (2025) 9:172. doi: 10.1038/s41698-025-00911-y 40500315 PMC12159146

[148] Wang L Wu H Wang J Han L Gu C Guo A . TRIM27 promotes ovarian cancer progression through destabilizing AMPK and thus inactivating the cGAS/STING signaling pathway. Int J Biol Macromol. (2025) 321:146268. doi: 10.1016/j.ijbiomac.2025.146268 40706920

[149] Zhang J Chen Y Chen X Zhang W Zhao L Weng L . Deubiquitinase USP35 restrains STING-mediated interferon signaling in ovarian cancer. Cell Death Differ. (2021) 28:139–55. doi: 10.1038/s41418-020-0588-y 32678307 PMC7853139

[150] Chowdhury SR Parikh CN Kaur AN DeMarco KD Giwa HK Mishra AK . PPT1 is a negative regulator of STING signaling in cancer cells and its inhibition reactivates immune surveillance in cold tumors. Proc Natl Acad Sci USA. (2025) 122:e2514948122. doi: 10.1073/pnas.2514948122 40982692 PMC12501185

[151] Griffin GK Wu J Iracheta-Vellve A Patti JC Hsu J Davis T . Epigenetic silencing by SETDB1 suppresses tumour intrinsic immunogenicity. Nature. (2021) 595:309–14. doi: 10.1038/s41586-021-03520-4 33953401 PMC9166167

[152] Yang H Sui L Cai C Chu H Diao Y . SETDB1 promotes progression through upregulation of SF3B4 expression and regulates the immunity in ovarian cancer. J Ovarian Res. (2024) 17:34. doi: 10.1186/s13048-024-01358-8 38317200 PMC10840244

[153] Miranda A Pattnaik S Hamilton PT Fuss MA Kalaria S Laumont CM . N-MYC impairs innate immune signaling in high-grade serous ovarian carcinoma. Sci Adv. (2024) 10:eadj5428. doi: 10.1126/sciadv.adj5428 38748789 PMC11095474

[154] Ye Z Xu S Shi Y Cheng X Zhang Y Roy S . GRB2 stabilizes RAD51 at reversed replication forks suppressing genomic instability and innate immunity against cancer. Nat Commun. (2024) 15:2132. doi: 10.1038/s41467-024-46283-y 38459011 PMC10923831

[155] Stojanovic L Abbotts R Tripathi K Coon CM Rajendran S Abbasi Farid E . ZNFX1 functions as a master regulator of epigenetically induced pathogen mimicry and inflammasome signaling in cancer. Cancer Res. (2025) 85:1183–98. doi: 10.1158/0008-5472.can-24-1286 39804147 PMC11968244

[156] Xie W Zhang L Shen J Lai F Han W Liu X . Knockdown of CENPM activates cGAS-STING pathway to inhibit ovarian cancer by promoting pyroptosis. BMC Cancer. (2024) 24:551. doi: 10.1186/s12885-024-12296-5 38693472 PMC11064423

[157] Bao Y Chang Y Tien JC Cruz G Yang F Mannan R . CDK12/13 inactivation triggers STING-mediated antitumor immunity in preclinical models. J Clin Invest. (2025) 135:e193745. doi: 10.1172/jci193745 40955658 PMC12435847

[158] Fang C Zhou S Yu K Zhang L Lian W Tie X . Lactate metabolism and lactylation in cancer: from pathogenesis to therapeutic advances. Signal Transduction Targeted Ther. (2026) 11:190. doi: 10.1038/s41392-026-02672-x 42161914 PMC13190781

[159] Certo M Tsai C-H Pucino V Ho P-C Mauro C . Lactate modulation of immune responses in inflammatory versus tumour microenvironments. Nat Rev Immunol. (2021) 21:151–61. doi: 10.1038/s41577-020-0406-2 32839570

[160] Lei Y VanPortfliet JJ Chen Y-F Bryant JD Li Y Fails D . Cooperative sensing of mitochondrial DNA by ZBP1 and cGAS promotes cardiotoxicity. Cell. (2023) 186:3013–3032.e22. doi: 10.1016/j.cell.2023.05.039 37352855 PMC10330843

[161] Dosta P Cryer AM Prado M Artzi N . Bioengineering strategies to optimize STING agonist therapy. Nat Rev Bioeng. (2025) 3:660–80. doi: 10.1038/s44222-025-00337-y37880705

[162] Sun X Zhang Y Li J Park KS Han K Zhou X . Amplifying STING activation by cyclic dinucleotide–manganese particles for local and systemic cancer metalloimmunotherapy. Nat Nanotechnol. (2021) 16:1260–70. doi: 10.1038/s41565-021-00962-9 34594005 PMC8595610

[163] Dane EL Belessiotis-Richards A Backlund C Wang J Hidaka K Milling LE . STING agonist delivery by tumour-penetrating PEG-lipid nanodiscs primes robust anticancer immunity. Nat Mater. (2022) 21:710–20. doi: 10.1038/s41563-022-01251-z 35606429 PMC9156412

[164] Wang S Chen N Bian W Liu T Xu X Xiao Y . Discovery of a non-nucleotide STING agonist with antitumor activity. Results Chem. (2026) 27:103456. doi: 10.1016/j.rechem.2026.10345642574925

[165] Meng Y Huang J Ding J Zhou H Li Y Zhou W . Mn-phenolic networks as synergistic carrier for STING agonists in tumor immunotherapy. Mater Today Bio. (2024) 26:101018. doi: 10.1016/j.mtbio.2024.101018 38516172 PMC10952078

[166] Liu Z Zhou J Gao R Wang Y Liu M Wang W . Precision-engineered STING agonist nanoparticles enable coordinated mucosal-systemic immunity for durable pan-β-coronavirus protection. Nat Nanotechnol. (2026) 21:1008–21. doi: 10.1038/s41565-026-02188-z 42310425

[167] Fu H Liu X Fang F Pan Z Chen L Chen G . Amplification of cGAS-STING pathway with “single-molecule multitarget” nanoparticles for chemo-immunotherapy of ovarian cancer. Biomaterials. (2025) 323:123434. doi: 10.1016/j.biomaterials.2025.123434 40446710

[168] Casadidio C Fens MHAM Fliervoet LAL Censi R Vermonden T . Injectable thermosensitive hydrogel for local and controlled delivery of siRNA-STAT3 polyplexes to treat advanced-stage ovarian cancer. J Controlled Release. (2025) 384:113890. doi: 10.1016/j.jconrel.2025.113890 40436346

[169] Zeng W Li Z Huang Q Ding C Yang L Wang W . Multifunctional mesoporous polydopamine-based systematic delivery of STING agonist for enhanced synergistic photothermal-immunotherapy. Adv Funct Mater. (2024) 34:2307241. doi: 10.1002/adfm.20230724142587375

[170] Zhong X Pu T Cheng Y Li Y Wang Q Wang B . Folic acid-modified antigen-trapping nanoprobes for developing in situ tumor vaccines to inhibit metastasis and recurrence of ovarian cancer. J Nanobiotechnol. (2026) 24:620. doi: 10.1186/s12951-026-04537-5 42116044 PMC13335270

[171] Agyemang A Wadhwa N Desouki M Bshara W Yu H Battaglia S . Intrinsic STING expression inversely associates with survival in ovarian cancer patients. Gynecologic Oncol. (2021) 162:S192. doi: 10.1016/s0090-8258(21)01013-1

[172] Konstantinopoulos P Moore KN Sachdev JC Mita MM Vinayak S Seward SM . Phase I/II study of niraparib plus pembrolizumab in patients with triple-negative breast cancer or recurrent ovarian cancer (KEYNOTE-162). J Clin Oncol. (2016) 34:TPS5599–TPS5599. doi: 10.1200/jco.2016.34.15_suppl.tps559942697810

